# Mechanisms of leading edge protrusion in interstitial migration

**DOI:** 10.1038/ncomms3896

**Published:** 2013-12-05

**Authors:** Kerry Wilson, Alexandre Lewalle, Marco Fritzsche, Richard Thorogate, Tom Duke, Guillaume Charras

**Affiliations:** 1 London Centre for Nanotechnology, University College London, 17-19 Gordon Street, London WC1H 0AH, UK; 2 Department of Physics and Astronomy, University College London, London WC1E 6BT, UK; 3 Department of Cell and Developmental Biology, University College London, London WC1E 6BT, UK; 4 Present address: Department of Chemistry, Imperial College London, London SW7 2AZ, UK; 5 Deceased

## Abstract

While the molecular and biophysical mechanisms underlying cell protrusion on two-dimensional substrates are well understood, our knowledge of the actin structures driving protrusion in three-dimensional environments is poor, despite relevance to inflammation, development and cancer. Here we report that, during chemotactic migration through microchannels with 5 μm × 5 μm cross-sections, HL60 neutrophil-like cells assemble an actin-rich slab filling the whole channel cross-section at their front. This leading edge comprises two distinct F-actin networks: an adherent network that polymerizes perpendicular to cell-wall interfaces and a ‘free’ network that grows from the free membrane at the cell front. Each network is polymerized by a distinct nucleator and, due to their geometrical arrangement, the networks interact mechanically. On the basis of our experimental data, we propose that, during interstitial migration, medial growth of the adherent network compresses the free network preventing its retrograde movement and enabling new polymerization to be converted into forward protrusion.

## Abstract (web summary)

Much of our understanding of the role of actin in cell migration is based on studies of cells moving across two-dimensional surfaces. Wilson *et al.* show that cells crawling in three dimensions through a narrow channel form two functionally distinct actin networks at the leading edge.

## Graphical abstract

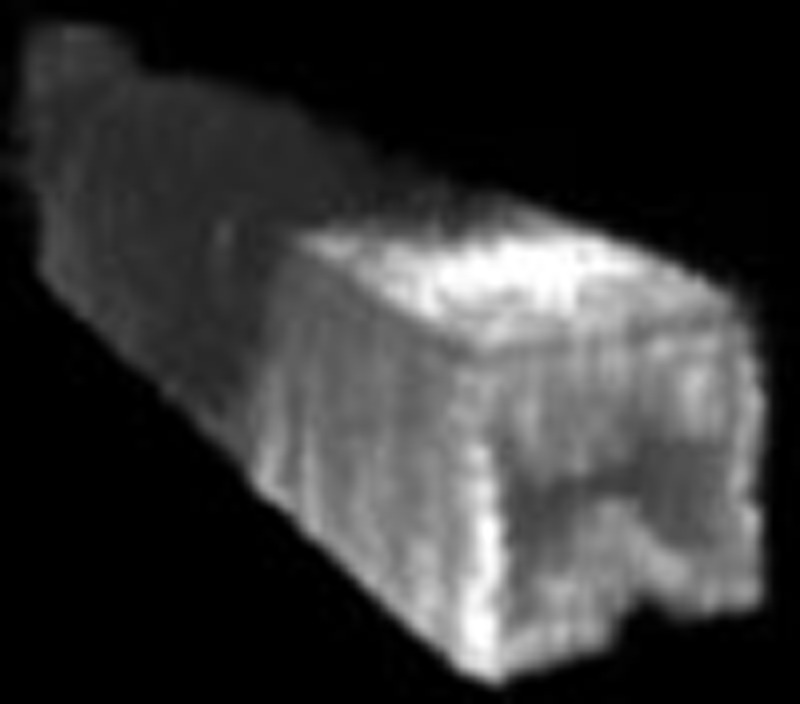

One of the most remarkable properties of animal cells is their ability to migrate. For experimental convenience, most research to date has concentrated on cell migration on two-dimensional (2D) planar surfaces. Although this has been pivotal to our present understanding of cell migration, many cell types migrate primarily in 3D environments: during development, cells move within the embryo to reach their correct location and, in disease, cancer cells leave the primary tumour to metastasize^1^. In particular, leukocytes circulate in the blood stream and upon entering an area of inflammation attach to the endothelium, traverse it, and migrate through tissues to reach the site of infection^2^,^3^. To carry out their immune function, they must move through tissues with many different organizations (from isotropic gels in mammary connective tissue to highly ordered collagen bundles running parallel to one another in the skin) and squeeze through gaps ranging from 2 to 10 μm in diameter^4^. Recent studies have made it increasingly apparent that migration in 3D environments differs in several key aspects from 2D migration. Indeed, whereas integrin-mediated adhesion to a substrate and myosin contractility are crucial for movement on planar substrates, they are not indispensable in confined and 3D environments^5^,^6^,^7^,^8^, underlining the limits of 2D models for understanding migration in physiologically relevant conditions.

Protrusion of the cell front is an essential step to migration and, in 2D, its mechanisms are now well understood both at the molecular level and at the biophysical level. On 2D substrates, migrating cells assemble lamellipodia, ~200 nm thick F-actin-rich veils, at their leading edge to protrude. Most incorporation of actin monomers takes place against the plasma membrane at the leading edge^9^,^10^ and actin filaments are organized in a dendritic network with their barbed-ends pointing towards the cell front through activation of the arp2/3 complex by WAS Family proteins^9^,^10^. From a biophysical standpoint, it is generally thought that oriented filament growth provides the force for forward protrusion of the cell membrane^10^. In contrast to the ubiquity of lamellipodia in 2D, in 3D environments, cells generate a variety of protrusions at their front: blebs^8^,^11^, filopodia, ruffle-like structures^6^, actin-rich lobopodia^12^ and pseudopodia^13^. Furthermore, cells can switch protrusion types during migration, spontaneously^8^ or in response to drug treatments^6^,^14^, and the choice of protrusion is thought to depend on the balance between actin polymerization, rear contractility and adhesion^8^,^15^. Previous work has examined the requirement for rear contractility^6^,^16^, and adhesion^6^, but our current understanding of even the most basic aspects of the actin dynamics underlying frontal protrusion in 3D remains poor.

Here we study leading edge protrusion during chemotactic migration of HL60 neutrophil-like cells by mimicking a 3D environment using microfluidic channels. The channels have cross-sections similar in dimension to the gap diameters leukocytes encounter during intravascular crawling, transmigration and migration through connective tissues^4^. In microchannels, the leading edge of migrating cells consists of an actin-rich slab several microns thick filling the whole channel cross-section and composed of two distinct F-actin networks that interact mechanically to give rise to forward protrusion. One network polymerizes perpendicular to cell-wall interfaces (the adherent network) and the other grows from the free membrane at the cell front (the free network). Polymerization of the free network is dependent upon the arp2/3 complex but formation of the adherent network is not, suggesting that each network results from polymerization by distinct nucleators. Removal of the free network by arp2/3 inhibition leads to a switch in mode of protrusion with the formation of blebs at the leading edge but does not inhibit migration.

## Results

### Neutrophil migration in microchannels

**Figure 1:**
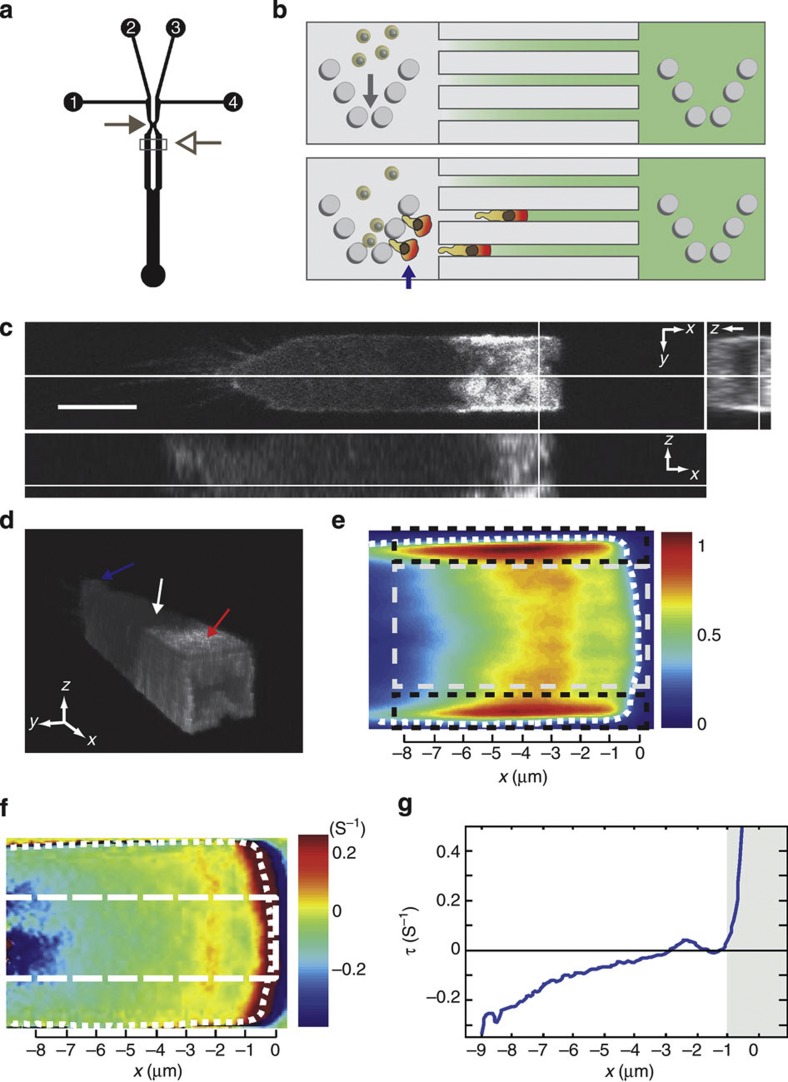
HL60 cells migrating in confined environments possess an F-actin-rich leading edge. In all panels, the front of the cell was taken as the origin of the *x* axis. (**a**) Microfluidic device for studying interstitial migration. The microfluidic device comprised four inlets (1–4) and one outlet. Cells were introduced into the device using inlet 2. Inlets 1 and 3 are used to circulate control medium (HBSS+BSA) and medium containing chemoattractant (fMLP), respectively. Both streams were brought into contact briefly (grey arrow) to equilibrate pressure before being separated for the creation of linear gradients (open arrow). Downstream, transversal channels with a 5 μm × 5 μm cross-section separated control stream from chemoattractant stream and interstitial cell migration was imaged in these (see **b**). (**b**-top) A linear gradient of chemoattractant was created between the control stream (grey, left) and the chemoattractant stream (green, right). This stimulated chemotaxis and migrating cells completely occluded the transversal channels (**b**-bottom, Supplementary Fig. S1B), a property that allowed selective treatment of the leading edge with drug (Supplementary Fig. S1C–E). (**c**) Orthogonal views of actin distribution in a live HL60 cell migrating in a microchannel visualized with GFP-actin. Scale bar=6 μm. (**d**) Perspective view of the cell in (**c**). The leading edge was surrounded by an interfacial region highly enriched in actin (red arrow). The nucleus and the uropod are indicated by the white and blue arrows, respectively. (**e**) Steady-state actin fluorescence intensity distribution in the leading edge *I*_*SS*_*(x,y)* in a plane midway through the channel height. Two regions could be distinguished on the basis of their fluorescence intensity: an adherent F-actin network situated at the interface between the cell and the channel walls (black boxed area) and a ‘free’ F-actin network situated in the inner part of the leading edge (grey boxed area). The dotted white line delineates the cell contour. (**f**) Net rate of change in actin density τ in the leading edge for the cell shown in **e**. Warm colours indicate increases in actin density and cold colours indicate decreases. The dotted line indicates the cell contour. (**g**) Net change in actin density τ as a function of distance from the cell front for the cell in **e**. The profile was calculated by averaging data in the boxed region in **f**. The greyed area indicates regions close to the front membrane.

In our experiments, we utilized neutrophil-like HL60 cells because their chemotaxis has been extensively characterized in 2D^17^ and because they have been shown to efficiently transmigrate^18^. To study migration through interstices, we examined chemotactic movement through microfluidic channels^13^ (Fig. 1a,b, Supplementary Fig. S1A) with cross-sections (~5 μm × 5 μm) within the range encountered *in vivo*^4^. Channels were functionalized with human serum albumin to allow adhesion of differentiated HL60 cells to channel walls via binding of α_M_β_2_ integrins (CD11b/CD18 and Mac-1)^19^. Migrating cells completely occluded the microchannels enabling application of drugs restricted to their leading edge (Supplementary Fig. S1B–E, (ref. 13)). Migration depended on myosin contractility, as previously reported^6^,^16^ and cells assembled a contractile actin cortex enriched in the membrane-actin linker protein moesin (Supplementary Fig. S2A) and myosin regulatory light chain (Supplementary Fig. S2B) at the cell rear. Importantly, cells migrated with a steady velocity and a highly constrained shape^13^, in contrast to natural environments where shape, velocity and directionality display considerable temporal variations^6^. Hence, microchannels offered an ideal environment to study the steady-state actin dynamics underlying protrusion in 3D.

### Migrating cells assemble an actin-rich slab at their front

Cells migrating through interstices possessed a leading edge highly enriched in F-actin (Supplementary Movie 1) but, in contrast to the thin protrusions observed in 2D, F-actin filled the entire channel cross-section forming a slab (Fig. 1c). In 3D perspective views of the cell, actin could clearly be distinguished lining all four channel walls in the leading edge (red arrow, Fig. 1d). When imaged in a horizontal cross section (*xy* plane) through the centre of the channel, the steady cell shape and motion during migration enabled computation of spatial maps of the steady-state F-actin fluorescence intensity (or equivalently density) *I*_SS_*(x,y)* and of the net instantaneous relative rate of change in F-actin density τ*(x,y)* (‘net rate of change in F-actin density’, see Supplementary Methods). The leading edge could be subdivided into two distinct regions based on F-actin density (Fig. 1e): (1) a dense network at the interface between the cell and channel walls (the ‘adherent’ F-actin network) and (2) a less dense region situated in the centre of the leading edge (the ‘free’ F-actin network). In the free network, F-actin density increased approximately twofold in the first 4 μm behind the free membrane at the cell front before decreasing thereafter (Fig. 1e). The net rate of change in F-actin density τ was maximum at the free front membrane (*x*=0 μm, Fig. 1f,g, 6/6 cells examined), suggesting that, as in 2D migration, most net actin assembly took place against the leading edge membrane (greyed zone, Fig. 1g, (refs 20, 21, 22, 23)). A second peak of net F-actin increase was visible ~2–3 μm behind the front membrane spanning most of the cell width (*x*=−2 μm, Fig. 1f,g, 6/6 cells examined), something that could be due to increased polymerization in that location or complex F-actin network flow patterns within the leading edge. Finally, net decrease in F-actin density occurred for locations further than ~2–3 μm behind the front membrane with a rate that appeared to increase with distance (Fig. 1g).

### The leading edge consists of two distinct F-actin networks

Given the distinctive morphology of the leading edge in 3D and the complex spatial pattern of net rate of change in F-actin density τ*(x,y)*, we investigated actin turnover and nucleation dynamics in 3D protrusions using fluorescence recovery after photobleaching (FRAP) experiments. In 2D, new F-actin polymerization against the membrane gives rise to a combination of forward protrusion and retrograde flow of the F-actin network relative to the substrate. Their relative importance has generally been formulated in the framework of the clutch hypothesis^24^: if the actin network is uncoupled from adhesion, no new protrusion is created and all polymerization is converted into retrograde flow (for example, in stationary cells^25^), and at the other extreme, if the actin network is perfectly coupled to the substrate, all polymerization is converted into protrusion. Most motile cells display a combination of retrograde flow and forward protrusion^26^,^27^.

**Figure 2:**
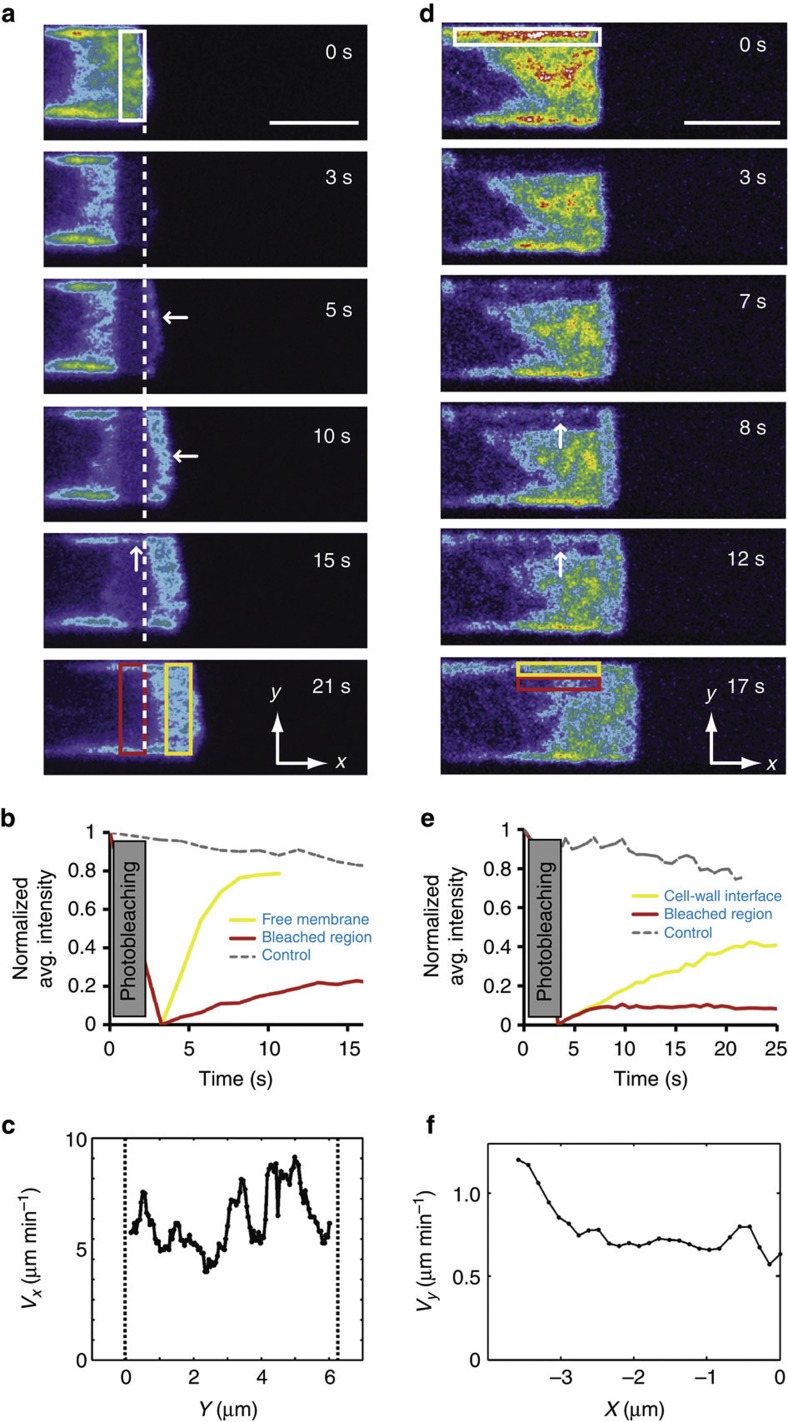
F-actin is nucleated in two separate regions within the leading edge. In all images, cold colours indicate low fluorescence intensities, and hot colours denote high actin fluorescence intensities. White indicates saturation in the chosen colour scale but does not reflect saturation during acquisition. Scale bars=5 μm. (**a**) FRAP experiment for a region including the free membrane at the front of a migrating cell expressing GFP-actin. The white box at *t*=0 s indicates the photobleached region. In all panels, the white dashed line indicates the position of the free front membrane at time *t*=0 s. (**b**) Time course of fluorescence recovery in the photobleached region and a region following the free front membrane. Fluorescence recovery was monitored in the bleached region, reflecting actin turnover (red box in **a** for *t*=21 s; red line, ‘bleached region’). For comparison, fluorescence increase arising from polymerization against the free membrane was measured in a region of identical size that followed the cell front over time (final position shown by yellow box in **a**, *t*=21 s; yellow line, ‘free membrane’). Fluorescence loss due to image acquisition was estimated in a ROI away from the bleach zone (grey line, ‘control’). (**c**) Polymerization velocity *v*_*x*_*(y)* at the free membrane at the cell front along the *y* axis for the cell shown in **a**. See Supplementary Fig. S4A–C for details. (**d**) FRAP experiment for a region at the cell-wall interface in the leading edge of a migrating cell expressing GFP-actin. The white box at *t*=0 s indicates the photobleached region. (**e**) Time course of fluorescence recovery at the cell-wall interface for the cell in panel **d**. Fluorescence was measured in a region tracking the bleached zone as it moved medially reporting on actin turnover (red box in **d** for t=17 s; red line, ‘bleached region’) or in a region that stayed stationary at the cell-wall interface that reflected new polymerization (yellow box in **d** for *t*=17 s; yellow line, ‘cell-wall interface’). Fluorescence loss due to image acquisition was estimated in a ROI away from the bleach zone (grey line, ‘control’). (**f**) Polymerization velocity *v*_*y*_*(x)* at the cell-wall interface along the *x* axis calculated from iFRAP data. The front of the cell was taken as the origin of the *x* axis. See Supplementary Fig. S4D–F for details.

In microchannels, when we photobleached a 2 μm × 5 μm rectangular region comprising the whole width of the free membrane at the cell front in a plane midway through the channel height (white box, Fig. 2a), fluorescence first reappeared in a thin band just outside of the bleached region in the direction of motion (horizontal arrows, *t*=5 s and *t*=10 s, Fig. 2a, Supplementary Movie 2), reflecting new protrusion created by F-actin polymerization against the free front membrane. New protrusion occurred approximately uniformly across the front membrane at rates *v*_*x*_~5.9±2.1 μm min^−1^, reflecting spatially uniform polymerization (6/6 cells observed, Fig. 2c, Supplementary Fig. S4A–C, Supplementary Methods). Over time, the bleached region did not undergo any detectable motion relative to the substrate, indicating the absence of retrograde flow and suggesting that all polymerization was converted into forward protrusion (10/10 cells observed, dashed line, Fig. 2a). Qualitatively similar results were obtained in inverse FRAP (iFRAP) experiments where the whole cell was bleached except for a small region around the free front membrane (Supplementary Fig. S3A). In 2D, most actin polymerization takes place against the leading edge membrane but some also occurs behind it^20^,^28^. To gain insight into the relative importance of polymerization at the free membrane and within the free F-actin network away from the membrane, we compared fluorescence recovery in the immobile bleached region that reflects filament turnover (red box, Fig. 2a; red line, Fig. 2b) to fluorescence recovery in an identically sized region tracking the free membrane and reporting on polymerization driving new protrusion (yellow box, Fig. 2a; yellow line, Fig. 2b). Over long time-scales, the final extent of fluorescence recovery *I*_∞_ in the bleached region was smaller and occurred slower (*I*_∞_=39±26% (mean±s.d.), *t*_1/2_=9.6±4.7 s, *N*=5 cells) than recovery at the free membrane (*I*_∞_=66.9±19.8%, *t*_1/2_=7.5±2.1 s, *N*=5 cells), indicating that most polymerization occurred at the free membrane and suggesting that the second peak of net increase in F-actin density observed ~2 μm behind the leading edge was not due to locally enhanced polymerization (Fig. 1f,g).

At longer times, localized fluorescence recovery was observed at the cell-wall interface within the bleached region, suggesting the presence of polymerization in this location (vertical arrow, *t*=15 s, Fig. 2a). Consistent with this, when we photobleached a 7 μm × 1 μm rectangular region at the cell-wall interface in the leading edge, actin fluorescence first recovered within the bleached region at the cell-wall interface (*t*=8 s, Fig. 2d, Supplementary Movie 3) and new polymerization displaced the bleached region towards the cell midline along the *y* axis, likely due to the rigid channel walls that prevented outward protrusion. Qualitatively similar results were obtained in iFRAP experiments (Supplementary Fig. S3B) and analysis of the medial movement of the adherent network revealed that polymerization velocity along the cell-wall interface *v*_*y*_*(x)* was slower than at the free membrane (*v*_*y*_~0.9±0.2 μm min^−1^, *N*=7 cells) with a front to rear gradient (Fig. 2f, Supplementary Fig. S4D–F, Supplementary Methods). Comparison of the fluorescence recovery at the cell-wall interface (due to new polymerization; yellow box, Fig. 2d) to recovery within an identically sized region moving along with the bleached zone and reporting on filament turnover (red box, Fig. 2d) revealed that new polymerization at the cell-wall interface was faster and more extensive than turnover away from it (Fig. 2e). To directly compare the rate of polymerization at the cell-wall interface and at the free front membrane, we monitored fluorescence recovery in identically sized regions that were either stationary at the cell-wall interface or tracked the free front membrane in experiments such as displayed on Fig. 2a (Supplementary Fig. S5). These paired measurements revealed that polymerization at the free front membrane was significantly faster than polymerization at the cell-wall interface (*t*_1/2, cell-wall interface_/*t*_1/2, free membrane_=1.7±0.8, *P*<0.01 compared with 1 with a *z*-test, *N*=7 cells, Supplementary Fig. S5B).

Together, these results revealed that actin polymerization took place predominantly in two locations in the leading edge: first, at the free membrane at the cell front (free F-actin network) where polymerization was faster and oriented in the direction of motion and second, at the cell-wall interface (adherent F-actin network) where polymerization was slower and directed outwardly, perpendicular to the channel wall.

### The actin networks in the leading edge interact mechanically

**Figure 3:**
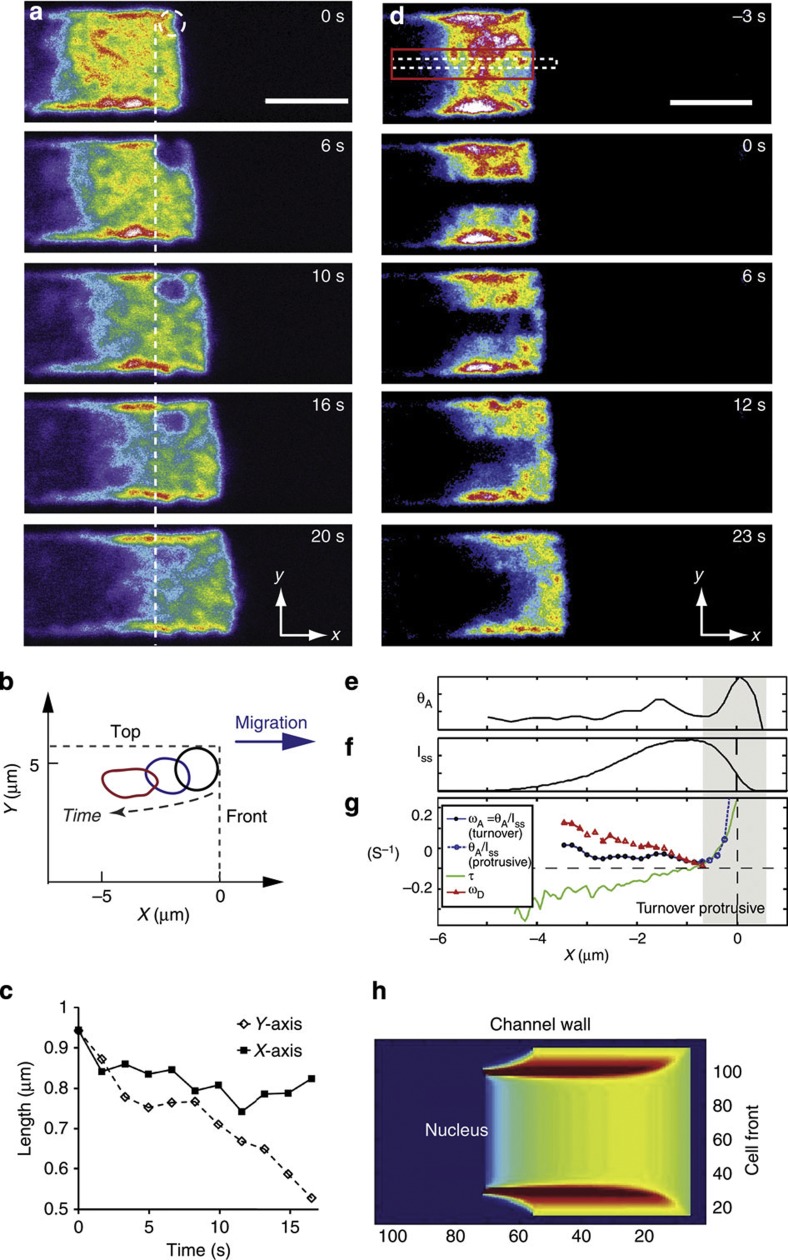
Mechanical interaction between the adherent and free networks and F-actin turnover dynamics in the leading edge. In all panels, the front of the cell at the time of photobleaching was taken as the origin of the *x* axis. All cells expressed actin-GFP. In all images, cold colours indicate low fluorescence intensities, and hot colours denote high actin fluorescence intensities. White indicates saturation in the chosen colour scale but did not reflect saturation during acquisition. Scale bars=5 μm. (**a**) FRAP experiment for a circular zone at the interface between the free membrane at the cell front and the cell-wall interface. Photobleaching was effected in the dashed white circle shown for *t*=0 s. On all panels, the white dashed line shows the rear of the bleach zone at *t*=0 s. (**b**) Change in shape of the bleached zone shown in **a** over time. (**c**) Change in horizontal (*x*) and vertical (*y*) axis lengths over time for the bleached region shown in **a**. (**d**) FRAP experiment for a rectangular region situated at the cell midline and spanning the whole length of the leading edge in a migrating cell expressing GFP-actin. Photobleaching was effected in the red box shown for *t*=0 s. Fluorescence recovery was monitored in a smaller box (dashed white box, *t*=0 s). (**e**) Rate of fluorescence increase *θ*_A_*(x)* at the midline determined experimentally for the cell shown in **d**. (**f**) Steady-state fluorescence intensity profile *I*_*SS*_*(x)* at the midline determined experimentally for the cell shown in **d**. (**g**) F-actin assembly rate constant *θ*_A_*(x)/I*_*SS*_*(x)* (blue line), net change in F-actin density τ (green line), and F-actin disassembly rate constant ω_*D*_ (red line) as a function of distance from the free front membrane for the cell shown in **d**. In regions of new protrusion (greyed area), *θ*_A_*(x)/I*_*SS*_*(x)* reported on new filament nucleation and elongation of existing filaments with their free barbed-ends abutting the membrane (dotted blue line and open symbols). In regions further from the front membrane, *θ*_A_*(x)/I*_*SS*_*(x)* was equal to the actin assembly rate constant ω_*A*_ and reflected G-actin incorporation through molecular processes participating in F-actin turnover (solid blue line and closed symbols). See Supplementary Fig. S7 for details on intermediate steps and calculations. (**h**) Theoretical actin fluorescence intensity distribution in the cell leading edge obtained from numerical simulations. See Supplementary Fig. S8 for details.

Following our initial experiments, we hypothesized that, as a consequence of their relative geometrical arrangement, the medial growth of the adherent F-actin network (Fig. 2d, Supplementary Fig. S3B) might compress the free F-actin network thereby preventing its retrograde flow and allowing new polymerization at the free membrane to generate protrusion. To test this experimentally, we followed the displacement and deformation of a small circular photobleached zone with a 1 μm radius at the interface between the free membrane and the channel wall (Fig. 3a). As the cell moved forward, the bleached region stayed stationary along the *x* axis but progressively moved medially along the *y* axis (Fig. 3a,b, Supplementary Movie 4). Concurrently, its shape changed from circular to ellipsoidal with its short axis along the *y* axis (Fig. 3b,c). The *x* axis length of the ellipsoid did not decrease significantly (0.8±0.2 μm, *P*=0.03 compared with initial diameter with a Student’s *t*-test, *N*=8 cells); whereas the *y* axis length displayed a significant ~50% decrease (0.5±0.1 μm, *P*<0.01 compared with initial diameter with a Student’s *t*-test, *N*=8 cells, Fig. 3b). Such a shape change could either be due to compressive stresses generated by medial growth of the adherent F-actin network or shear stresses arising from differential movement of the adherent and free networks along the *x* axis, for example. Pure compression should result in an increase in F-actin density, whereas pure shear should not. Steady-state F-actin density maps displayed a twofold increase in density ~3–4 μm behind the front membrane (Fig. 1e), consistent with the length-scale over which the bleach zone changed shape (Fig. 3b), and our FRAP experiments indicated only weak polymerization away from the free front membrane (Fig. 2a,b). Together, these data suggested that the change in aspect ratio of circular bleach regions was due to compression by medial growth of the adherent network. In support of this, iFRAP experiments indicated a global decrease in height of the free network along the *y* axis over time (−22±5%, *N*=6 cells, Supplementary Fig. S3A,C) coupled to medial displacement of the adherent network (Supplementary Fig. S3B,D).

### Actin assembly is maximal behind the free front membrane

In 2D, studies in several different cell types revealed that a net increase in F-actin density occurs immediately behind the front membrane owing to nucleation of new filaments by the arp2/3 complex and incorporation of G-actin monomers at the free barbed-ends of existing filaments abutting the front membrane; while a net decrease takes place further away from the membrane^20^,^21^,^22^,^23^. The respective contributions of F-actin assembly and disassembly to these net changes in F-actin density have been estimated using quantitative speckle microscopy^20^,^23^. In 2D protrusion, F-actin assembly peaks ~1 μm behind the membrane due to nucleation, before decreasing to a plateau for distances larger than ~1.5 μm^20^. In contrast, disassembly is most active ~1.2 μm from the membrane but less active and constant for distances greater than 1.5 μm. We investigated whether the similar profiles of net rate of change in F-actin density τ*(x)* observed in 2D and 3D protrusions were reflective of similar actin dynamics.

To determine the rate of F-actin assembly in 3D protrusions, we photobleached 7 μm × 2 μm rectangular regions located midway through the cell height, at the cell midline and spanning the whole leading edge length (red box, Fig. 3d, Supplementary Movie 5). We then monitored fluorescence recovery in a smaller region contained within the bleach region to minimize any potential contribution from F-actin network flows along the *y* axis (dashed white box, Fig. 3d). Because there was no retrograde flow relative to the substrate along the *x* axis and because there was no flow convergence at the midline, the initial rate of fluorescence increase *θ*_A_*(x)* at each location *x* reflected the instantaneous rate of incorporation of fluorescent G-actin monomers (Supplementary Methods). *θ*_A_ *(x)* was maximal just behind the front membrane before decreasing with distance away from the leading edge (Fig. 3e, Supplementary Fig. S7C, Supplementary Methods). To interpret these data, we distinguished a protrusive regime and a turnover regime for G-actin incorporation. First, in regions of new protrusion, we expect G-actin incorporation to be high due to the high local density of free barbed-ends necessary to achieve forward protrusion of the membrane through polymerization together with the nucleation of new filaments. Second, in regions further away from the membrane, we expect free barbed-ends to exist in finite proportion relative to the local F-actin density, regulated by the processes that participate in the turnover of F-actin. On the basis of its localization within a region of new protrusion (Fig. 3d; greyed zone, Fig. 3e), we associated the maximum in *θ*_A_*(x)* occurring for 0.5 μm ≥*x*≥−0.5 μm with the protrusive regime (Fig. 3e). Regions further away were situated within the bleached region (*x*≤−0.5 μm) and therefore we concluded that *θ*_A_*(x)* in these regions reflected the turnover regime (Fig. 3e). Within regions undergoing turnover, the G-actin monomer incorporation rate *θ*_A_*(x)* is the product of the local F-actin density and the G-actin monomer incorporation rate per unit mass of F-actin *ω*_A_*(x)* (or assembly rate constant, in s^−1^) that reflects, for example, the local density of free barbed-ends. To determine which of these two variables caused the spatial variations in *θ*_A_*(x)* within the bleached region (Fig. 3e), we determined *ω*_A_*(x)* by normalizing *θ*_A_*(x)* to the steady-state actin fluorescence intensity along the midline *I*_SS_*(x)* (Fig. 3f, Supplementary Methods). For regions in the turnover regime (*x*≤−0.5 μm), *ω*_A_(x) was constant signifying that the rate of G-actin monomer incorporation per unit mass of F-actin was constant, perhaps due to a constant density of free barbed-ends (5/5 cells examined, Fig. 3g, solid blue line with closed symbols), and therefore spatial variations in the monomer incorporation rate *θ*_A_*(x)* were wholly imputable to spatial variations in the steady-state F-actin density *I*_SS_*(x)*.

For interpretation of our experimental data, we formally defined the net rate of change in F-actin density *τ* as *τ(x)=ω*_A_*(x)−ω*_D_*(x)+sδ(x)* with *ω*_A_ the F-actin assembly rate constant due to turnover processes, *ω*_D_ the F-actin disassembly rate constant and *s*δ*(x)* a source term with *s* its amplitude and δ*(x)* the Dirac function. In this framework, the rates *ω*_A_ and *ω*_D_ were only defined in regions where the fluorescence intensity before photobleaching was non-zero. The source term reflected new protrusion generated by F-actin nucleation and incorporation of G-actin monomers into existing filaments with their free barbed-ends located at the front membrane. Because of the limited spatiotemporal resolution of optical microscopy and due to irregularity in the outline of the front membrane, the contribution of the source term *sδ(x)* to τ*(x)* appeared broadened in regions of new protrusion with a width ~0.5 μm (Fig. 1g; green line, Fig. 3g) and the area under the curve should in principle reflect the amplitude *s*. In practice, determination of the source term through division of *θ*_A_*(x)* by *I*_SS_ wa*s* unreliable near the membrane due to the small absolute value of *I*_SS_ (Fig. 3f and dotted blue line with open symbols, Fig. 3g). However, in graphs of *θ*_A_*(x)*, the source term translated into a bell-shaped curve with a height on average three- to fourfold larger than for regions in the turnover regime, thereby confirming that monomer incorporation occurs predominantly, but not only, at the front membrane (Fig. 3e). Lack of retrograde flow coupled to cell movement at a constant velocity *v* enabled determination of the net rate of change in F-actin density τ*(x)* (green line, Fig. 3g) from the experimentally determined steady-state actin fluorescence intensity along the midline *I*_SS_*(x)* (Fig. 3f): *τ(x)=−v*[*∂I*_SS_*(x)/∂x*]/*I*_SS_*(x)*. As the source term was zero away from the membrane, the disassembly rate constant *ω*_D_ could then be determined as *ω*_D_*(x)=ω*_A_*(x)−*τ*(x)* (Supplementary Fig. S7, Supplementary Methods). This revealed that *ω*_D_ increased with distance from the membrane (5/5 cells examined, Fig. 3g, red line), signifying that monomers were lost more rapidly per unit mass of F-actin.

To determine whether actin polymerization at the cell front and at the cell-wall interface could lead to the kinematics of actin flow (Figs 2 and 3, Supplementary Fig. S3) and actin densities observed in the leading edge (Fig. 1e), we generated a simple computational model of actin dynamics incorporating: polymerization at the channel walls and at the free front membrane (Fig. 2) and lack of interpenetration and material exchange between the two F-actin networks (Supplementary Fig. S6). In this framework, when the disassembly rate *ω*_D_ increased with distance from cell boundaries as suggested by our experiments (Fig. 3g), we were able to replicate the experimentally observed steady-state F-actin densities (Figs 1e and 3h, Supplementary Fig. S8D), but, when *ω*_D_ was taken constant, simulations could not qualitatively replicate the observed actin density profile (Supplementary Fig. S8C, Fig. 1e). Together, these results show that actin polymerization is highest at the leading edge membrane and is proportional to F-actin density throughout the rest of the leading edge, whereas actin depolymerization increases away from the leading edge membrane.

### The free actin network is nucleated by the arp2/3 complex

**Figure 4:**
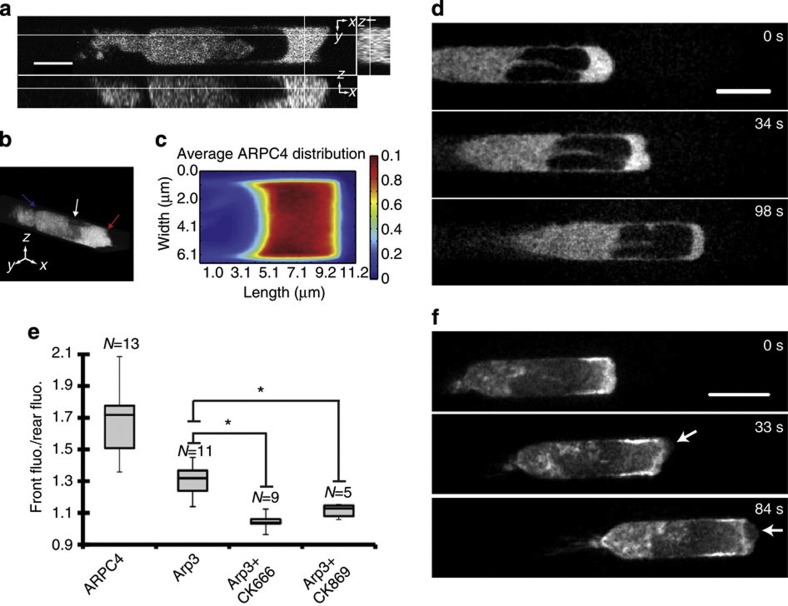
Arp2/3-mediated nucleation generates the free F-actin network but is not necessary for migration. (**a**) Localization of the ARPC4 subunit of the arp2/3 complex in live migrating HL60 cells. Orthogonal views of ARPC4 distribution in cells migrating in confined environments. Scale bar=6 μm. (**b**) Perspective view of the cell in **a**. The leading edge, nucleus and uropod are indicated by red, white and blue arrows, respectively. (**c**) Steady-state ARPC4 distribution in the leading edge. (**d**) Local application of the arp2/3 inhibitor CK666 to the leading edge of a cell expressing ARP3-GFP. CK666 was applied exclusively to the leading edge at *t*=0 s. Scale bar=5 μm. (**e**) Ratio of frontal fluorescence intensity to rear fluorescence intensity for cells expressing ARPC4, ARP3 and ARP3 in the presence of CK666, and ARP3 in the presence of CK869. Data are plotted as box-whisker plots in which the top of the box represents the first quartile of the data, the bottom the third quartile and the line denotes the median. Whiskers indicate the maximum and minimum measurements. The number of cells for each measurement is indicated above each box. Asterisks denote significant differences when comparing populations pairwise with a Student’s *t*-test (*P*<0.01). (**f**) Local application of the arp2/3 complex inhibitor CK666 to the leading edge of a cell expressing actin-GFP. CK666 was applied exclusively to the leading edge at *t*=0 s. Blebs at the leading edge resulting from CK666 treatment are shown by white arrows. Scale bar=10 μm.

Next, we sought to understand the molecular mechanisms generating the free and adherent F-actin networks. During chemotaxis in 2D, the phospholipid PI-(3,4,5)P_3_ (PIP3) accumulates at the cell front in a feedback loop involving PI(3)-kinase and the small GTPase Rac that leads to polymerization of actin through WAVE complex-mediated activation of the arp2/3 complex^29^,^30^. Similarly, in interstitial migration, the PIP3 reporter PH-Akt localized to the membrane in the whole of the leading edge (Supplementary Fig. S2D), consistent with observations in neutrophils migrating in live zebrafish^31^. In contrast, the non-specific membrane marker MyrPalm localized uniformly throughout the whole cell membrane (Supplementary Fig. S2C). Interestingly, whereas the ARP3 and ARPC4 subunits of the arp2/3 complex were clearly localized to the lamellipodium during 2D migration (Supplementary Fig. S9, Supplementary Movie 7), they displayed uniform localization to the leading edge in interstitial migration (Fig. 4a–c, Supplementary Fig. S2E, Supplementary Movie 6). However, both subunits were clearly enriched in the leading edge with average ratios between front and rear fluorescence intensities of *r*_ARP3_=1.3±0.1 (*N*=11 cells) for ARP3 and *r*_ARPC4_=1.7±0.2 (*N*=13 cells) for ARPC4 (Fig. 4a–c,e, Supplementary Fig. S2E, Supplementary Movie 6).

To investigate how F-actin was polymerized in 3D protrusions, we applied chemical inhibitors of polymerization solely to the cell leading edge using our microfluidic device (Supplementary Fig. S1B–E, (ref. 13)). In these experiments, cells are initially stimulated to migrate with a chemoattractant gradient (green, Supplementary Fig. S1C-top; Supplementary Fig. S1D: *t*=0 s). Then, the medium bathing the leading edge is replaced within ~45 s by medium containing chemoattractant, a chemical inhibitor and a different fluorophore to track medium exchange kinetics (blue, Supplementary Fig. S1C**-**bottom, Supplementary Fig. S1D: *t*=50 s, Supplementary Fig. S1E). As a control, we first treated the leading edge of migrating cells with the actin filament barbed-end capper cytochalasin D. This led to complete cessation of movement and protrusion within 60 s (15/15 cells observed, Supplementary Fig. S1F, Supplementary Movie 8), confirming that actin polymerization was necessary. Next, we examined the role of arp2/3-mediated nucleation by perturbation with the small-molecule inhibitor CK666 (ref. 32) that leads to loss of lamellipodia within 60 s and complete inhibition of chemotactic movement in 2D (Supplementary Fig. S10B). During migration in microchannels, application of CK666 to the cell front caused a significant loss in enrichment in ARP3 in the leading edge (*r*_CK666_=1.06±0.07, *N*=9 cells, *P*<0.01 when compared with control conditions with a Student’s *t*-test), consistent with reports examining *Aplysia* growth cones^23^ (Fig. 4d,e). A similar effect was also noted with the structurally unrelated arp2/3 inhibitor CK869 (ref. 32) (*r*_CK869_=1.11±0.04, *N*=5 cells, *P*<0.01 when compared with control conditions with a Student’s *t*-test, Fig. 4e). For both inhibitors, loss in Arp2/3 complex enrichment was accompanied by a switch in protrusion type to blebbing in ~50% of the cells examined (18/34 cells examined, Fig. 4d,f, Supplementary Movie 9) with the remainder displaying no marked phenotype, perhaps due to incomplete inhibition. In cells that switched to blebbing, the free F-actin network disappeared but the adherent actin network appeared unaffected (Fig. 4f, *t*=84 s). Surprisingly, migration velocity was accelerated by arp2/3 inhibition (*v*_CK666_=9.3±1.8 μm min^−1^, *N*=4 cells; *v*_Control_=4.9±1.0 μm min^−1^, *N*=8 cells; *P*<0.01 when compared with a Student’s *t*-test). These data indicate that polymerization of the free F-actin network is dependent on the arp2/3 complex and that the free network is dispensable for motility.

**Figure 5:**
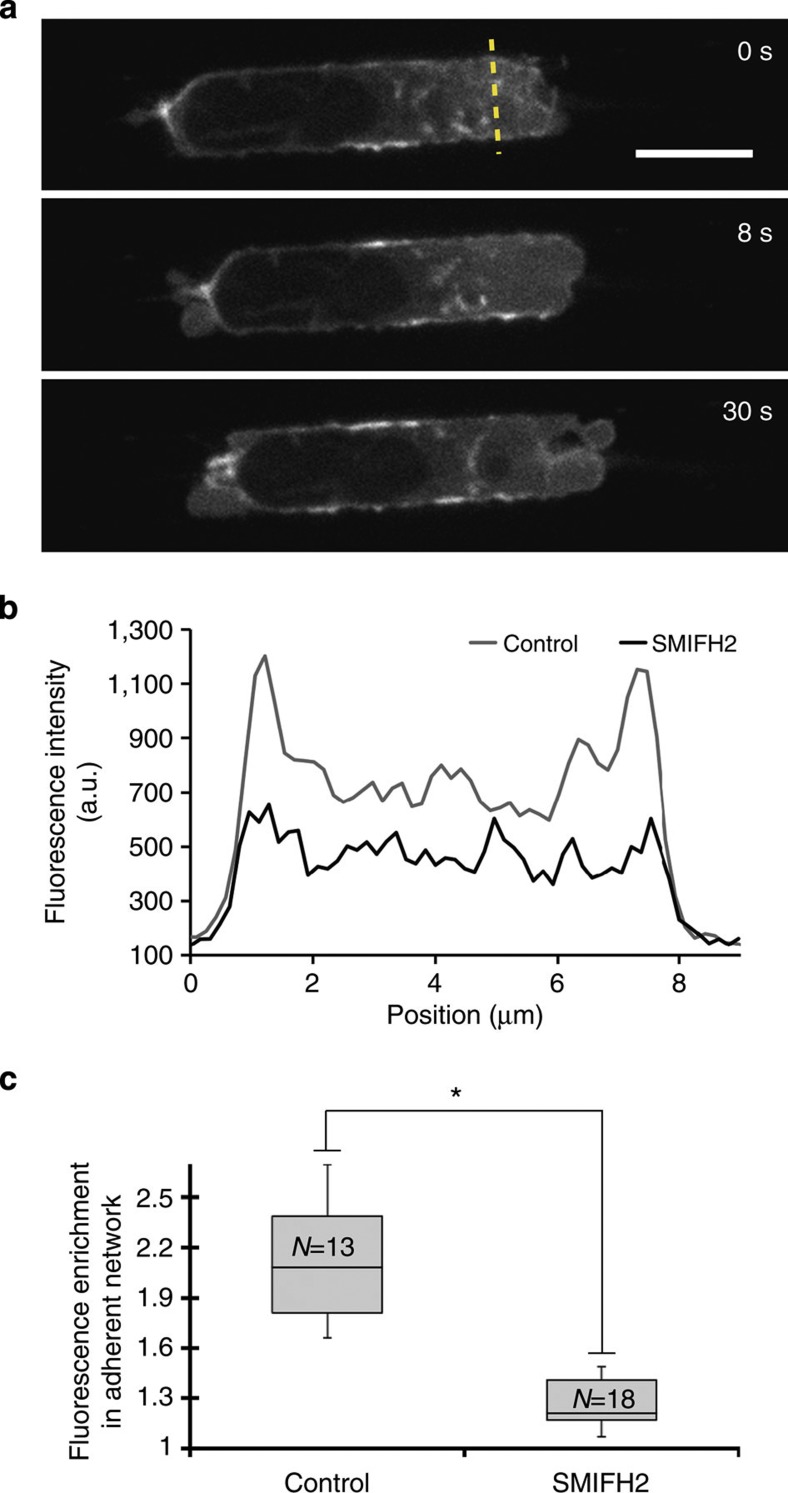
Formin-mediated polymerization generates the adherent F-actin network and is necessary for migration. (**a**) Local application of the formin inhibitor SMIFH2 to the leading edge of a cell expressing actin-GFP. SMIFH2 was applied exclusively to the leading edge at *t*=0 s. Scale bar=5 μm. (**b**) Fluorescence intensity profile across the leading edge for a line situated 2 μm behind the free front membrane for a control cell and a cell treated with SMIFH2 (yellow dotted line in **a**). (**c**) Fluorescence enrichment in the adherent F-actin network in control cells and cells treated with SMIFH2. Data are plotted as box-whisker plots in which the top of the box represents the first quartile of the data, the bottom the third quartile and the line denotes the median. Whiskers indicate the maximum and minimum measurements. The number of cells for each measurement is indicated within each box. The asterisk denotes a significant difference when comparing both populations with a Student’s *t*-test (*P*<0.01).

The lack of arp2/3 complex enrichment in the adherent F-actin network (Fig. 4a, Supplementary Fig. S2E) together with resistance of the adherent network to CK666 treatment suggested that it might be nucleated by formins. Indeed, previous work in 2D migration has shown the presence of formin-mediated actin polymerization in the leading edge^33^,^34^. Treatment with small-molecule inhibitor of formin homology 2 domains (SMIFH2, a broad spectrum inhibitor targeting the FH2 domain of formins^35^, led to rapid cessation of movement in 2D (Supplementary Fig. S10C). When applied locally to the cell front in microchannels, SMIFH2 led to a loss in the F-actin enrichment observed in the adherent network (Fig. 5a–c), destabilized leading edge morphology (Fig. 5a, Supplementary Movie 10) and halted migration (Fig. 5a, 17/17 cells examined, Supplementary Movie 10). These data taken together suggest that both formins and the arp2/3 complex participate in nucleation of F-actin in leading edge protrusions of HL60 cells migrating in 3D.

## Discussion

**Figure 6:**
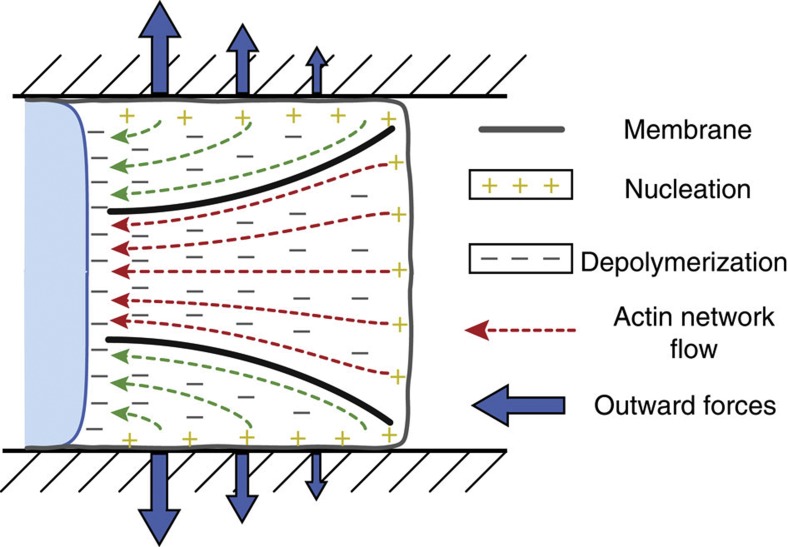
Conceptual model of F-actin dynamics in leading edge protrusion during interstitial migration. In confined environments, leading edge protrusion results from the interaction between the adherent and the free F-actin networks. Nucleation of the adherent network (green) is directed perpendicular to the channel walls and its thickness increases from front to rear creating a constriction that compresses the free F-actin network (red). This constriction prevents rearward movement of the free F-actin network enabling new polymerization under the front membrane to create forward protrusion. Compression of the free network by the adherent network creates an outward pressure on the channel walls (arrows) as well as a protrusive force at the free membrane.

Here we examined actin dynamics in the leading edge of differentiated HL60 cells during interstitial migration. On the basis of actin density, turnover and chemical inhibition, we were able to distinguish two functionally distinct F-actin networks in these protrusions, an adherent network and a free network. The free F-actin network had a lower F-actin density, faster F-actin assembly and was polymerized by the arp2/3 complex against the free membrane at the cell front. The adherent network polymerized against the cell-wall interface was enriched in F-actin compared with the free network and was not affected by arp2/3 inhibition. Interestingly, inhibition of formins blocked migration suggesting that, like in 2D, formin-mediated F-actin nucleation is important for migration^33^,^34^. Together, these data suggest a picture of protrusion in microchannels where the adherent F-actin network grows medially from the interface with the channel walls via formins and surrounds the free F-actin actin network that polymerizes from the free membrane at the cell front via arp2/3 nucleation (Fig. 6). Remarkably, the functional differences observed between the adherent and free actin networks in 3D protrusions mirrored in several points the distinctions drawn between the lamellipodium and the lamellum in the leading edge of cells migrating in 2D. Indeed, the lamellipodium is nucleated by the arp2/3 complex^10^, has a faster F-actin assembly rate^20^, and is weakly adherent to the substrate^9^; whereas the lamellum is formed of longer less branched actin filaments nucleated by formins such as mDia2 (refs 33, 34), has a lower assembly rate^20^, and a stronger adhesion to the substrate^9^. Furthermore, recent studies have shown that cells can migrate in 2D without a lamellipodium^36^,^37^, similar to our observation that the free F-actin network is dispensable for migration in microchannels. Interestingly, when the free F-actin network was lost due to arp2/3 inhibition, cells continued migrating but switched to blebbing motility. Such a phenotypic change could either be due to an increase in rear contractility upon arp2/3 inhibition as reported in Walker carcinosarcoma cells^8^, to a loss of tethering between WAS family proteins in the front membrane and arp2/3 proteins in the free F-actin network (as suggested in the lamellipodium^38^,^39^,^40^) or a combination of both. Inhibition of formins with SMIFH2 led to cessation of movement but, due to the broad spectrum of this inhibitor, further experiments will be necessary to understand the exact role of formins in interstitial migration. Quantitative analysis of actin dynamics also revealed that migration in 2D and 3D differed in several key aspects. In 3D, net increases in F-actin density were observed in two locations in the leading edge: against the free membrane at the cell front and ~2 μm behind it, a pattern similar to what is observed in 2D^20^,^21^,^22^,^23^. However, whereas in 2D the second peak forms at the interface between the lamellipodium and the lamellum^20^,^23^, in 3D it occurs in the free F-actin network due to compression resulting from medial growth of the adherent F-actin network. In interstitial migration as in 2D migration, G-actin incorporation was maximal immediately behind the free front membrane where a dendritic network of F-actin was generated through arp2/3 nucleation. Then, for distances greater than 0.5 μm from the front membrane, the assembly rate constant *ω*_A_ was constant, qualitatively similar to the plateau observed for distances greater than ~1.5 μm from the front membrane in 2D^20^,^21^,^22^,^23^. However, in 3D, the disassembly rate constant *ω*_D_ increased steadily with distance from the membrane, in contrast to the constant rate reported for distances greater than ~1.5 μm in 2D^20^. This signifies that, in 3D, depolymerization becomes more active per unit mass of F-actin network as the distance to the front membrane increases, something that could have either a biochemical origin (such as preferential recruitment of cofilin to older ADP-actin rich filaments^41^) or a mechanical origin (such as a stimulation of depolymerization by increased compressive stresses at the rear of the leading edge^42^). In the present study, our observations were restricted to a neutrophil-like leukaemia cell line in stiff microfabricated channels and therefore it will be important to determine the generality of our findings in healthy and malignant cell types migrating through the softer interstices encountered in the human body.

Our experiments lead us to propose that, in confined microchannels, the geometrical arrangement of F-actin polymerization in the leading edge results in a mechanical interaction between the adherent and free F-actin networks that generates the mechanical forces necessary for protrusion. Indeed, a dendritic network of F-actin (the free network) was rapidly polymerized from the free membrane at the cell front with its barbed-end oriented in the direction of protrusion (Fig. 6, red). Simultaneously, another F-actin network (the adherent network) was polymerized perpendicular to the cell-wall interface and the stiff channel wall forced it to grow medially (Fig. 6, green). This resulted in an adherent network with a thickness that increased from front to rear, creating a constriction. Consequently, the free F-actin network was subjected to increasing compressive stresses, something that prevented its retrograde movement and enabled all new polymerization to be converted into protrusion. Using this simple picture, the magnitude of forces generated during protrusion in microchannels can be estimated assuming linear elasticity for the F-actin networks and an elastic modulus of ~1 kPa for dendritic actin gels^43^,^44^. Indeed, the measured change in aspect ratio of circular bleach regions in the direction perpendicular to motion (Fig. 3a–c, Supplementary Fig. S3) suggests that medial growth of the adherent network results in compressive stresses of up to ~500 Pa in the free network. Conversely, a similar magnitude of outward pressures must be exerted by the cell on the channel walls at the leading edge (Fig. 6, arrows), something suggested in physical models of dendritic cell migration^45^. Interestingly, the estimated magnitude of outward stresses should give rise to significant deformations in physiological environments, such as extracellular matrix gels. The resistance to lateral expansion of the adherent gel provided by the stiff channels may itself contribute to the protrusive mechanism in addition to the forces generated by forward-directed actin polymerization against the free front membrane. Indeed, the average strain tensor *ε*_*x*_ along the *x* axis in the free network is *ε*_*x*_*=(*σ_*x*_*−*νσ_*y*_*−*νσ_*z*_*)/E*, where *E* is the elastic modulus of the actin network, σ the stress tensor and ν the Poisson ratio of the actin network. Our experiments examining deformation of circular bleach regions suggested that the F-actin network was under little or no strain along the *x* axis (*ε*_*x*_~0). Therefore, assuming *σ*_*y*_*=σ*_*z*_*=−p*_wall_~500 Pa, the lateral compression of the free F-actin network by the adherent network generated an outward pressure *σ*_*x*_=−2*νp*_wall_~500 Pa for *ν*=0.5 (ref. 46), comparable to stall pressures of 1,600 Pa measured for primary neutrophils migrating in micropipettes^47^. Further work will be necessary to test this model experimentally and dissect the relative contributions of the free and adherent F-actin networks to forward protrusion as well as assess the relevance of the observed protrusion geometry for 3D cell migration *in vivo*.

## Methods

### Cell culture

The neutrophil-like human leukaemia cell line HL60 was a kind gift of Dr Orion Weiner (University of California in San Francisco). Cells were maintained in RPMI 1640 with Glutamax (Invitrogen), 10% fetal bovine serum and penicillin/streptomycin. Cells were passaged every 3 to 4 days to maintain cell concentration between 10^5^ and 10^6^ cells ml^−1^. Before experiments, cells were differentiated by adding 1.3% dimethylsulphoxide (DMSO) to the culture medium and left to differentiate for 3–5 days.

### Creation of stable cell Lines

To examine localization of cytoskeletal and signalling proteins during cell migration, we generated HL60 cell lines stably expressing proteins tagged with fluorescent proteins. Creation of all lines followed the same procedure: complementary DNA encoding the protein of interest tagged with fluorescent protein was excised from a donor vector and inserted into one of the retroviral vectors pRetroQAcGFPN1/C1, pLNCX2 or pLPCX (Takara-Clontech). Actin green fluorescent protein (GFP) was acquired from Clontech. Actin-mRFP was previously described^48^. Life-Act-mCherry was a kind gift of Dr Roland Wedlich-Söldner (MPI, Martinsried)^49^. Moesin-GFP was a kind gift from Prof Heinz Furthmayr (Stanford University). Myosin Regulatory Light Chain-GFP was described in Charras *et al.*^48^ PH-Akt-GFP was from Dr Tobias Meyer (Addgene plasmid 2121, (ref. 50)). P20/ARPC4 was a kind gift of Prof Klemens Rottner (University of Bonn). ARP3-GFP described in Charras *et al.*^48^ and was a kind gift from Prof Matt Welch (University of California Berkeley). Myristoylated GFP was from Daniel Gerlich (Addgene plasmid 21037, (ref. 51)). Retroviruses were then generated by transfecting these plasmids into 293-GPG cells for packaging (a kind gift from Prof Daniel Ory, Washington University^52^). Retroviral supernatants were then harvested and used to transduce wild-type HL60 cells.

For transduction with retrovirus, 5 × 10^4^ cells were centrifuged at 500 *g* for 3 minutes and resuspended in 1 ml of viral supernatant with 8 μl ml^−1^ polybrene. The resulting suspension was incubated at 37 °C for 5–6 h before a second round of transduction. After 2 to 3 days recovery, cells were selected with the appropriate antibiotic (1 μg ml^−1^ of puromycin or 1 mg ml^−1^ G418). Cultures remained under selection conditions until they reached ~10^6^ cells ml^−1^, after which time the cells were amplified for fluorescence-activated cell sorting (FACS).

### FACS sorting

In order to select only high expressors of GFP-tagged proteins, cells were selected using FACS. Briefly, 5 × 10^7^ cells were collected, resuspended in 10 ml of Gey’s balanced salt solution (Sigma) and passed through a cell strainer. Cells were sorted using a Beckman Coulter MoFlo XPD system with a 488 nm argon laser for fluorescence activation. To collect cells with high levels of fluorescence, only the top 2% highest expressing cells were collected. The sorted cells were then resuspended in a 1:1 mixture of normal and preconditioned RPMI. Cells were then amplified for use in microscopy experiments.

### Cell migration in microfluidic devices

Before experimentation, the devices were passivated for 1 h with a solution of Hank’s Balanced Salt Solution containing 1.8% human serum albumin. Tygon tubing (OD 0.762 mm, ID 0.254 mm, Universal Biologics, Cambridge, UK) was inserted into the inlets and outlets and interfaced with 15 ml conical vials containing the solutions to be used during experiments. Liquids were driven on chip using an MFCS-FLEX pressure regulation system (Fluigent, Paris, France). The tubing was primed with buffer solution before connecting to the device to prevent air bubbles from entering the microfluidic channels. Four 15 ml conical vials were prepared with buffer solutions used during experimentation (one with buffer only; one with buffer or cell suspension; one with buffer, chemoattractant and Alexa647-labelled dextran (Invitrogen, Paisley, UK); and one with buffer, chemoattractant, drug and Cascade Blue-labelled dextran (Invitrogen)). Fluid flow rates were adjusted using the software interface of the MFCS-FLEX system and allowed to equilibrate for several minutes. Once a stable flow was achieved, buffer containing cells was introduced via inlet 2 (Fig. 1a) and the fluid stream focused such that the cells were delivered to chevron-shaped pillar arrays that served to trap the cells (Fig. 1b**-**top). Once the arrays were saturated with cells, the fluid streams were adjusted such that a linear gradient of chemoattractant was created across the transversal channels (5 μm × 5 μm cross-section) connecting the chemoattractant stream to the control stream (Fig. 1b, control medium is shown in grey and medium containing chemoattractant in green). Upon sensing the chemoattractant, cells migrated out of the pillar arrays and into the transversal channels where they were imaged (Fig. 1b**-**bottom, arrow).

### Drug treatments in microfluidic migration assays

Local perfusion experiments were performed in our microfluidic devices under laminar flow conditions. Devices and cells were prepared and introduced as previously described, with the exception that the channel containing chemoattractant was split to include an additional fluid stream containing chemoattractant and a drug of interest (inlets 3 and 4 in Fig. 1a). Under initial conditions, the pressure on the fluid stream containing the drug (as visualized by Cascade Blue-labelled dextran, stream 4, blue Supplementary Fig. S1C-top) was low compared with the pressure on the fluid stream containing only chemoattractant (stream 3, green, Supplementary Fig. S1C-top). This ensured that the solution in stream 3 shielded cells migrating through transversal channels from drug (Supplementary Fig. S1C-top). Once sufficient cells had penetrated into the transversal channels and established sustained stable migration, the pressure on the fluid stream containing the drug (stream 4) was increased and the pressure on the stream containing chemoattractant only (stream 3) reduced to zero such that the drug containing solution could now diffuse into the transversal channels (Supplementary Fig. S1C-bottom). Contact of the drug containing medium with the cells was visualized using the cascade blue-labelled dextran included in the treatment stream (Supplementary Fig. S1D). The effect of the drug on the migrating cell was then imaged using time-lapse confocal microscopy.

### Imaging

Time-lapse microscopy imaging was effected using either a spinning disk confocal microscope or an Olympus FV-1000 scanning laser confocal microscope. All fluorescence imaging was performed using a × 100 oil immersion objective (UPlanSApo, NA=1.4, Olympus) on an inverted microscope (IX81, Olympus). The spinning disk confocal was fitted with a Yokogawa spinning disk head (Yokogawa, CSU22) interfaced to an iXon camera (Andor, Belfast, UK) and acquisition was piloted with iQ software (Andor). Fast acquisition of confocal image stacks was enabled by a Prior Nanoscanz stage (Prior Scientific, Cambridge, UK). Acquisition on the scanning laser confocal was piloted with the Olympus FV-ASV software. Excitation with a 488 nm wavelength laser was utilized for GFP-tagged proteins, with a 543 nm wavelength laser for RFP- and mCherry-tagged proteins, with a 405 nm wavelength laser for Cascade blue-labelled dextrans, and with a 647 nm wavelength laser for Alexa647-labelled dextrans.

### Photobleaching and iFRAP experiments

FRAP experiments were performed on HL60 cells expressing GFP-tagged actin migrating within the transversal microchannels. FRAP experiments were performed using a × 100 oil immersion objective lens (NA=1.4) on a scanning laser confocal microscope (Olympus Fluoview FV1000). Before experiments, we empirically determined the photobleaching exposure settings necessary to reduce fluorescence of a region of interest (ROI) to background level in cells expressing actin-GFP fixed with 4% paraformaldehyde (to preserve GFP fluorescence and prevent diffusive recovery). With our setup, exposure of a ROI within the leading edge of a migrating cell to 488 nm laser at 100% power (20 mW nominal power) for 2 s with an 8 μs per pixel dwell-time reduced fluorescence intensity to that of the background. Furthermore, use of a confocal microscope coupled with a high magnification objective restricted photobleaching to an optical section whose thickness was determined empirically to be 0.6 μm along the *z* axis. To avoid any potential out of plane contributions to fluorescence recovery due to flows of F-actin network along the *z* axis, photobleaching experiments were carried out in an *xy* plane midway through the height of the channel.

For FRAP experiments, the imaging protocol was the following: two frames were acquired for normalization of the fluorescence signal, then, the fluorescence was bleached with a single iteration of the bleach pulse, and finally recovery was imaged over several minutes. Recovery images were acquired at the highest achievable frame rate and they were used to monitor the displacement and fluorescence recovery of the ROI as the cell migrated through the channel. In iFRAP experiments, the whole cell was photobleached except for a small ROI.

For better visualization of fluorescence evolution at short times after photobleaching, time series are presented in pseudo-colour scales to enable regions of weak fluorescence to be clearly distinguished (Figs 2 and 3, Supplementary Figs S3–S7). In these images, pixels with intensities above the maximum of the chosen colour scale appear in white but this does not signify that pixels were saturated during acquisition of the fluorescence signal.

### FRAP analysis

Fluorescence recovery in the leading edge was either analysed manually or using custom written Matlab software. Following photobleaching, fluorescence in the ROI can recover either through diffusion of unbound fluorescent G-actin monomers (diffusive recovery) or through incorporation of fluorescent G-actin monomers into the F-actin network (reactive recovery). Recovery through diffusion is fast necessitating less than 250 ms for regions with their smallest dimension equal to 1 μm (see Supplementary Information in Fritzsche *et al.*^53^). As in our experiments, the first frame of recovery was collected 1 s after completion of photobleaching, we reasoned that diffusive recovery was complete by then and that, by subtracting the average fluorescence intensity in the ROI in the first recovery frame from subsequent time points, we could monitor reactive recovery alone. In some measurements, fluorescence intensities were then normalized to the initial fluorescence intensity minus the fluorescence intensity in the first post-bleach frame (or cytoplasmic fluorescence, Fig. 2b,e, Supplementary Fig. S5B) and recovery curves were plotted using Microsoft Excel. Midline photobleaching experiments (Fig. 3d, Supplementary Fig. S7) were analysed using custom written Matlab software described in detail in Supplementary Methods. To estimate fluorescence loss due to image acquisition, we measured the evolution of fluorescence intensity in control regions away from the ROI, subtracted background fluorescence intensity and normalized intensities to their initial value minus background intensity (Fig. 2b,e, Supplementary Fig. S5B). When we measured an apparent reaction rate constant *ω*_*fl*_ for fluorescence loss due to imaging with the same algorithms used to estimate the apparent assembly rate constant *ω*_*A*_, we found *ω*_*fl*_~0.003 s^−1^, approximately one order of magnitude smaller than the typical values measured for *ω*_*A*_.

### Statistical analysis

Differences in migration velocity upon chemical treatment, recovery half-times, ratios of front to rear fluorescence intensity and enrichment at the cell-wall interface were assessed with Student’s *t*-tests using Microsoft Excel. Values of *P*<0.01 were deemed statistically significant.

## Author contributions

K.W., T.D. and G.C. designed the study. K.W. designed and manufactured the microfluidic devices. K.W. performed the experiments. A.L., K.W. and M.F. analysed the data. M.F. and K.W. performed the FRAP experiments. A.L. and T.D. designed the numerical simulation. A.L. and M.F. contributed analytical tools. G.C. and R.T. contributed reagents. K.W., A.L. and G.C. wrote the paper.

## Additional information

**How to cite this article:** Wilson, K. *et al.* Mechanisms of leading edge protrusion in interstitial migration. *Nat. Commun.* 4:2896 doi: 10.1038/ncomms3896 (2013).

## Supplementary Material

Supplementary Figures and Supplementary Methods Supplementary Figures S1-S10 and Supplementary Methods

Supplementary Movie 1 F-actin localisation in a differentiated HL60 cell expressing Life-Act-mCherry migrating in a confined environment. Migrating cells possessed an F-actin rich leading edge at the front as well as a less enriched actin cortex at the rear. Scale bar 5 μm. Total duration 34 s.

Supplementary Movie 2 Photobleaching of the leading edge of a differentiated HL60 cell expressing GFP-actin during migration in a microchannel (corresponding to Fig 2A). Scale bar 5 μm. Total duration 25 s.

Supplementary Movie 3 Photobleaching of the cell-wall interface in a differentiated HL60 cell expressing GFP-actin during migration in a microchannel (corresponding to Fig 2D). Scale bar 5 μm. Total duration 25 s.

Supplementary Movie 4 Photobleaching of a circular region at the interface between the free membrane at the cell front and the cell-wall interface in a differentiated HL60 cell expressing GFP-actin during migration in a microchannel (corresponding to Fig 3A). Scale bar 5 μm. Total duration 25 s.

Supplementary Movie 5 Photobleaching of a rectangular region along the cell midline in a differentiated HL60 cell expressing GFP-actin during migration in a microchannel (corresponding to Fig 3D). Total duration 52 s.

Supplementary Movie 6 Localisation of the arp3 subunit of the arp2/3 complex during migration in a microchannel. Scale bar 5 μm. Total duration 25 s.

Supplementary Movie 7 Localisation of the ARPC4 subunit of the arp2/3 complex during migration in 2-D in a differentiated HL60 cell expressing GFP-ARPC4. The white circle indicates the tip of a micropipette filled with chemoattractant. Scale bar 5 μm. Total duration 250 s.

Supplementary Movie 8 Local application of cytochalasin D to the leading edge of migrating cells inhibits migration. The presence of cytochalasin D at the leading edge was visualised by inclusion of a blue fluorophore in the drug containing stream. Actin localisation was visualised in cells stably expressing mRFP-actin. Imaging was started immediately after application of cytochalasin to the leading edge. After 60s treatment, the leading edge was devoid of actin. Upon cessation of cytochalasin treatment, the cell restarted migration. Scale bar 5 μm. Total duration 200 s.

Supplementary Movie 9 Local application of the arp2/3 complex inhibitor CK666 does not inhibit migration (corresponding to Fig 4D). Actin localisation was visualised in cells stably expressing GFP-actin. The presence of CK666 at the leading edge was visualised by inclusion of a fluorophore in the drug containing stream (shown in red). Imaging was started immediately after application of CK666 to the leading edge. Following 30 s treatment, the cell switched to blebbing motility. Scale bar 5 μm. Total duration 160 s.

Supplementary Movie 10 Local application of the formin inhibitor SMIFH2 inhibits migration. Actin localisation was visualised in cells stably expressing GFP-actin. The presence of SMIFH2 at the leading edge was visualised by inclusion of a fluorophore in the drug containing stream (shown in blue). Imaging was started immediately after application of SMIFH2 to the leading edge. Treatment with SMIFH2 led to a loss of protrusion at the leading edge and inhibited migration. Scale bar 5 μm. Total duration 490 s.
