# Supplementary material for: Mechanisms of leading edge protrusion in interstitial migration
Source: Nat Commun. 2013 Dec 5;4:2896. doi: 10.1038/ncomms3896 (PMC3863902; doi:10.1038/ncomms3896)
Supplement: Supplementary Figures and Supplementary Methods — Supplementary Figures S1-S10 and Supplementary Methods [file ncomms3896-s1.pdf]

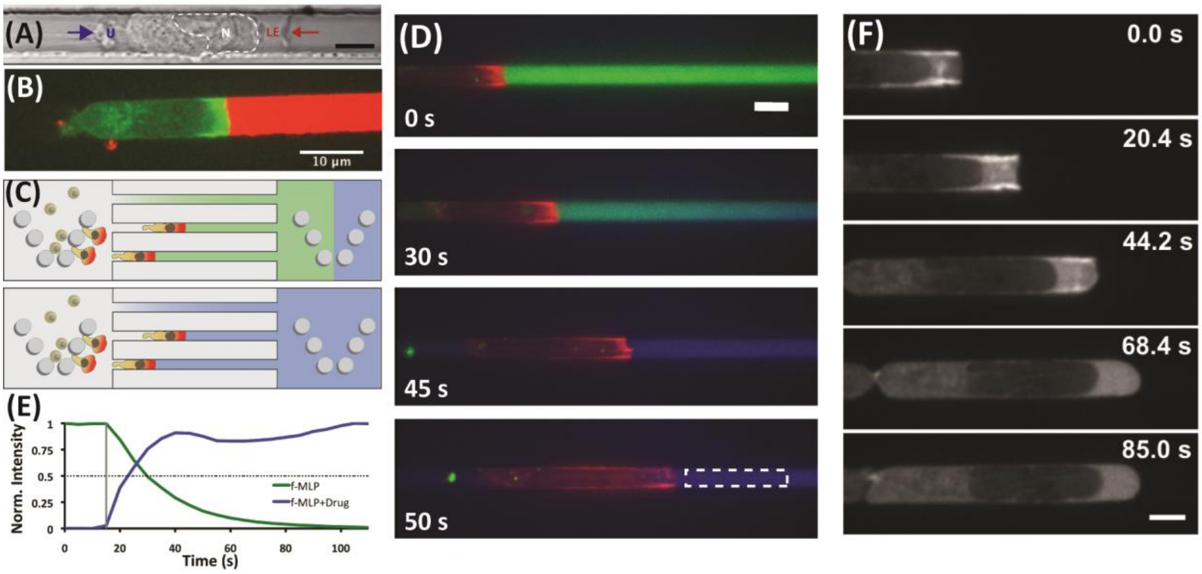

Supplementary Figure S1: Microfluidic devices enable local application of drug to the leading edge.

A. **Differential Interference Contrast image of a differentiated HL60 cell migrating through a transversal microchannel**. While migrating through the microchannel, cells adopted an elongated morphology with a short leading edge (LE) at the front, a contractile uropod at the rear (U), and were in close contact with the channel walls (visible at the top and bottom of the image). Nuclei appeared compressed between the walls (white dashed line, N). Scale bar = 5µm.
B. **Migrating cells completely occlude transversal microchannels.** A small fluorescent dextran (red) was included in the chemoattractant stream (stream 3, **Fig 1A**) and could not diffuse past a cell (green) migrating in the channel. Scale bar=10µm.
C. **Local treatment experiments.** Inlets 3 and 4 (**Fig 1A**) can be used for application of drugs to the leading edge of cells only. (**top**) Because of the laminar properties of fluid flow in microfluidic channels, when medium was circulated through inlet 3 (green, control medium + chemoattractant) and 4 (blue, medium + chemoattractant + inhibitor), both streams flowed side by side without mixing and therefore cells migrating in the transversal channels were shielded from exposure to inhibitor. (**bottom**) When stream 3 was turned off, it no longer shielded the cells from the inhibitor and the leading edge of migrating cells was now exposed to the inhibitor but not their rear, thereby creating a linear intracellular gradient of inhibitor.
D. **Time series of medium exchange at the leading edge.** At time *t*=0s, cells migrated through channels in response to a chemoattractant gradient that was visualised by addition of a green fluorophore to the medium in inlet 3. Medium containing chemoattractant, a drug, and a blue fluorophore was circulated through inlet 4 simultaneously but could not reach the cells because they were shielded by stream 3 (see C-top). At *t*=5s, flow through inlet 3 was turned off and stream 4 could now occupy the whole of the right hand channel (see C-bottom), progressively reaching the cells through diffusion (*t*=30, 45, 50s).
E. **Kinetics of medium exchange at the leading edge.** The graph shows the temporal evolution of the fluorescence intensity of control medium containing chemoattractant and a green fluorophore (green line) as well as the fluorescence intensity of medium containing chemoattractant, a drug, and a blue fluorophore (blue line). Fluorescence intensities were measured in the white box shown on panel D and were normalised to their maximal values measured over the duration of the time-lapse. At time *t*=15s, medium exchange was started. By 60s, the concentration of medium containing the blue fluorophore (blue) had reached

80% of its maximum concentration and the control medium was less than 20% of its initial concentration. We concluded that medium exchange necessitated ~45s to complete.

F. **Local application of cytochalasin D to the leading edge of a migrating cell.** Cytochalasin D was applied exclusively to the leading edge at *t*=0s. Scale bar=5µm.

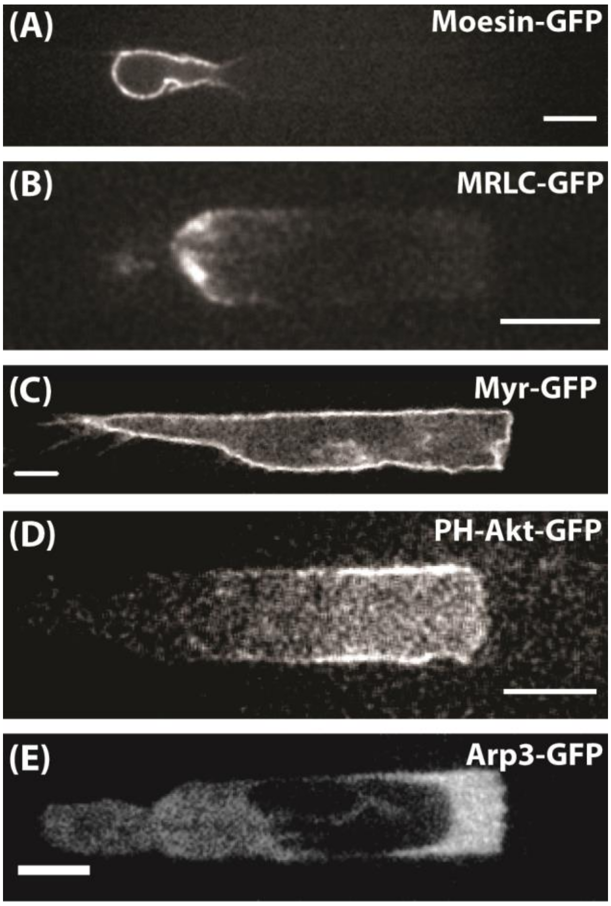

Supplementary Figure S2: Localisation of cytoskeletal and membranous proteins in live differentiated HL60 cells migrating in microchannels. In all panels, cells are moving from left to right. Scale bars = 5µm.

- **A. Moesin localisation in migrating cells.** The ERM protein moesin localised to the cell rear and was particularly enriched in the uropod of migrating cells.
- **B. Myosin localisation in migrating cells.** The regulatory light chain of myosin was polarised to the cell rear but appeared excluded from the uropod.
- **C. Membrane localisation in migrating cells.** Myristoylated GFP localised uniformly to the cell membrane.
- **D. PIP3 localisation in migrating cells.** The PH-domain of Akt localised to the F-actin rich cell front indicating the presence of an accumulation of PIP3 at the leading edge.
- **E. ARP3 localisation in migrating cells.** The ARP3 subunit of the arp2/3 complex localised to the cell front with the same distribution as the ARPC4 subunit (**Fig 4A**).

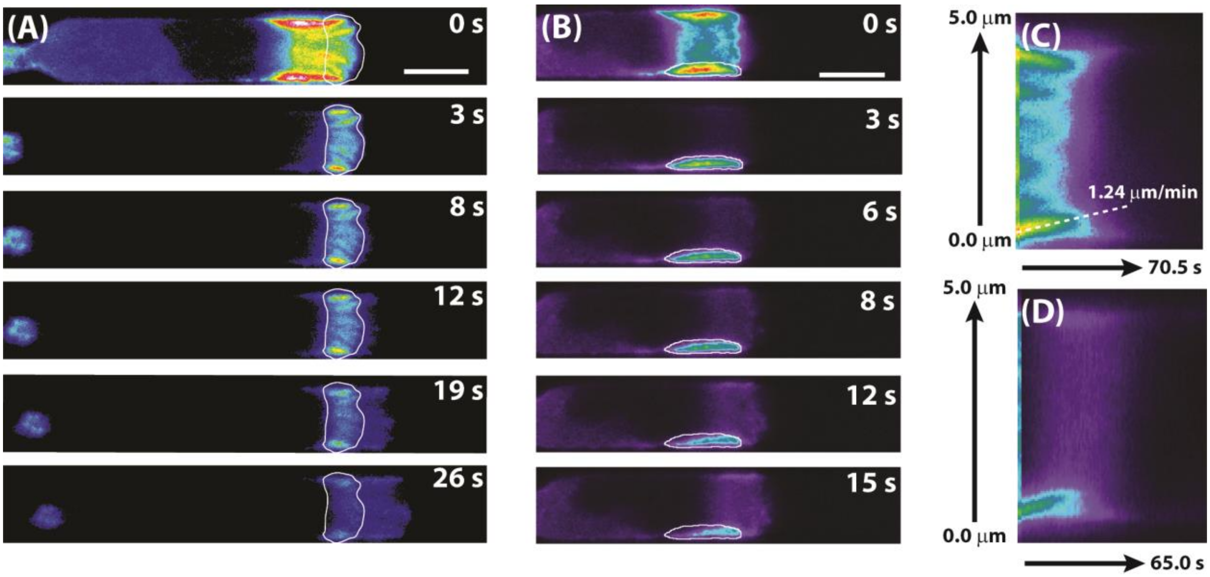

Supplementary Figure S3: Inverse FRAP (iFRAP) experiments in the leading edge of migrating differentiated HL60 cells. In all panels, actin localisation was visualised with GFP-actin. In all images, cold colours indicate low fluorescence intensities, and hot colours denote high actin fluorescence intensities. White indicates saturation in the chosen colour scale but does not reflect saturation during acquisition. Scale bars=5μm.

A. **Time series of an iFRAP experiment where only the cell front (free F-actin network) was not bleached.** On all frames, the dashed white lines indicate the shape of the cell front at *t*=0s. Immediately after acquisition of the image at *t*=0s, the whole cell except for the zone delineated by the white line was photobleached. Over time, the non-bleached zone stayed stationary with respect to the substrate along the *x*-axis, consistent with **Fig 2A**, but it appeared to decrease in dimension along the *y*-axis over time, consistent with **Fig 3A-C** and suggesting that it was subjected to increasing compression. Decrease in height could also clearly be visualised in kymographs such as the one presented in panel **C**. Over time, the fluorescence intensity of the non-bleached region decayed significantly, suggesting that the F-actin network contained within it was turning over, and it disappeared quasi-completely once it was located at the rear of the leading edge (*t*=26s).
B. **Time series of an iFRAP experiment where only the adherent F-actin network was not bleached.** On all frames, the dashed white lines indicate the shape of the adherent F-actin network at *t*=0s. Immediately after acquisition of the image at *t*=0s, the whole cell except for the zone delineated by the white line was photobleached. Over time, the non-bleached F-actin network stayed stationary with respect to the substrate along the *x*-axis but moved medially along the *y*-axis due to polymerisation of non-fluorescent F-actin against the cell-wall interface, consistent with **Fig 2D** and **Fig 3A-C**. Medial movement could also clearly be visualised in kymographs such as the one presented in panel **D**. Over time, the fluorescence intensity of the non-bleached region decayed significantly, suggesting that the F-actin network contained within it was turning over, and it progressively shortened in length as the cell leading edge moved forward (compare *t*=6s to *t*=15s).
C. **Kymograph of the temporal evolution of the height of the free F-actin network shown in A**. The kymograph was acquired along a vertical line through the centre of the non-bleached region. Over time, the height of the free actin network diminished. Height change was symmetrical with respect to the cell midline. The dashed line indicates the rate of height change. The velocity of inward movement of the free network was consistent with the measured polymerisation velocities v_y for the adherent network (**Fig 2F**).

D. Kymograph of temporal evolution of the position of the adherent F-actin network shown in B. The kymograph was acquired along a vertical line through the centre of the non-bleached region. The adherent network moved medially over time, suggesting that it was displaced by polymerisation of new non-fluorescent F-actin against the cell-wall interface. The velocity of medial movement of the adherent network was similar to the velocity of inward movement measured for the free network in **C**.

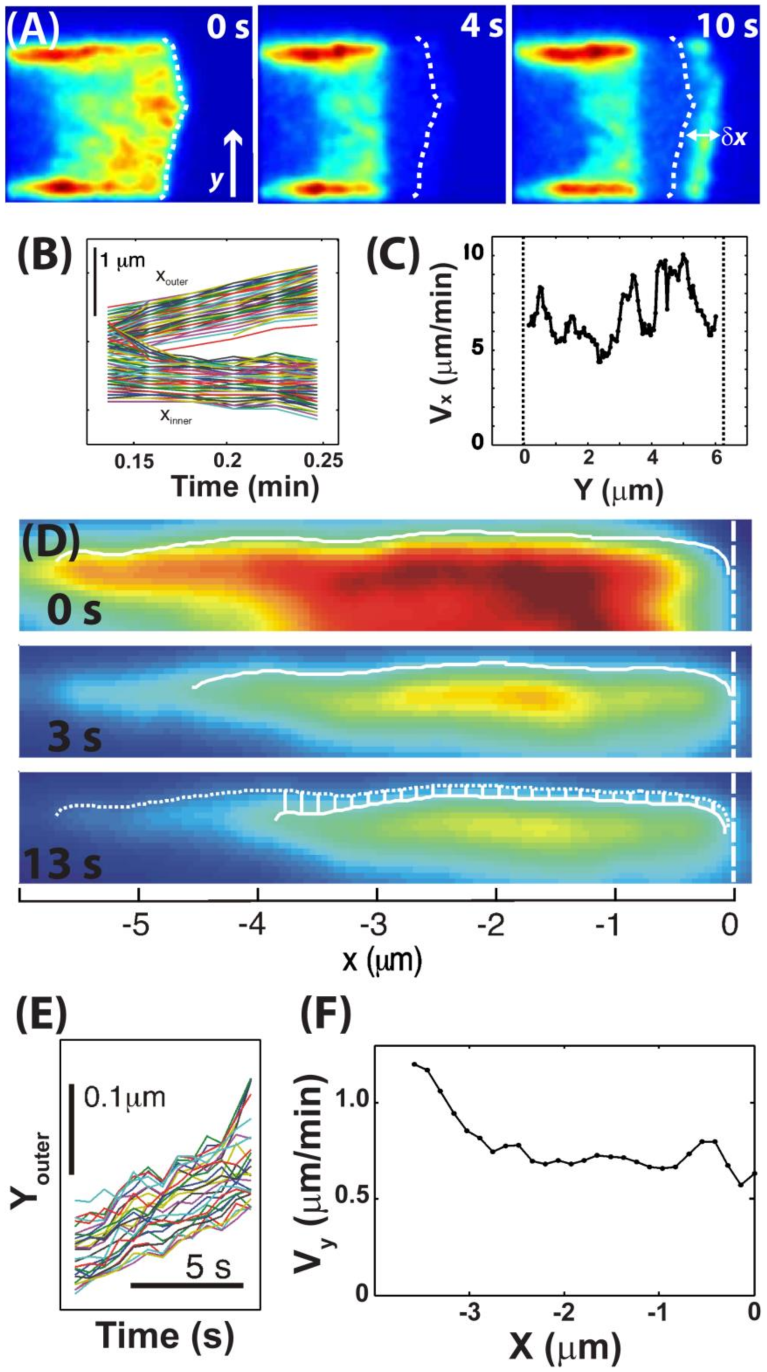

Supplementary Figure S4: Calculation of the spatial polymerisation velocity profiles at the free membrane and at the cell-wall interface. In all images, cold colours indicate low fluorescence intensities, and hot colours denote high actin fluorescence intensities.

A. **Time series of a FRAP experiment where only the free membrane at the cell front was bleached.** The first panel (*t*=0s) shows the actin fluorescence intensity in the cell leading edge prior to photobleaching. Immediately after photobleaching, no fluorescence could be observed in the cell front (*t*=4s). Following photobleaching, actin fluorescence recovered just outside of the bleached region due to new protrusion (*t*=10s). On all panels, the dashed white line indicates the position of the membrane at the cell front prior to photobleaching.
B. **Position of the inner and outer boundaries of the new protrusion as a function of time.** Using image segmentation, we determined the inner (x_{inner}) and outer (x_{outer}) boundaries of

the fluorescent region created by new protrusion ($\delta x(y)=x_{outer}(y)-x_{inner}(y)$ on panel **A,** *t*=10s) for all time points and at each pixel along the *y*-axis. Over time, x_{outer} increased linearly but x_{inner} stayed approximately constant, consistent with the absence of retrograde flow in the leading edge.

C. Spatial profile of the front membrane velocity. The evolution of δx with time was computed from the positions of x_{inner} and x_{outer} and this allowed computation of the polymerisation velocity v_x at each pixel along the *y*-axis.

D. Time series of an iFRAP experiment where only the adherent F-actin network was not bleached. Only the adherent F-actin network is displayed and the channel wall is located at the top of the image. The first panel shows the actin fluorescence intensity at the cell-wall interface prior to iFRAP (*t*=0s). The whole cell except for the adherent F-actin network at one of the walls was bleached (*t*=3s), as in **Supplementary Fig 3B**. Following photobleaching, the adherent F-actin network moved medially over time due to polymerisation of non-fluorescent F-actin against the cell-wall interface. In all panels, the position of the free membrane at the cell front at time *t*=0s is indicated by the dashed white line and it was chosen as the reference along the *x*-axis.

E. Position of the outer boundary of the adherent F-actin network over time. Using image segmentation, we determined the outer position of the adherent *F*-actin network at each time point (indicated by the white line on panels in **D**) and at each pixel along the *x*-axis.

F. Spatial profile of the adherent network velocity. The evolution of the position of the outer boundary of the adherent network at each pixel over time (**E**) allowed computation of the spatial profile of the velocity of the adherent network $v_y(x)$ that reflected new polymerisation of non-fluorescent F-actin against the cell-wall interface.

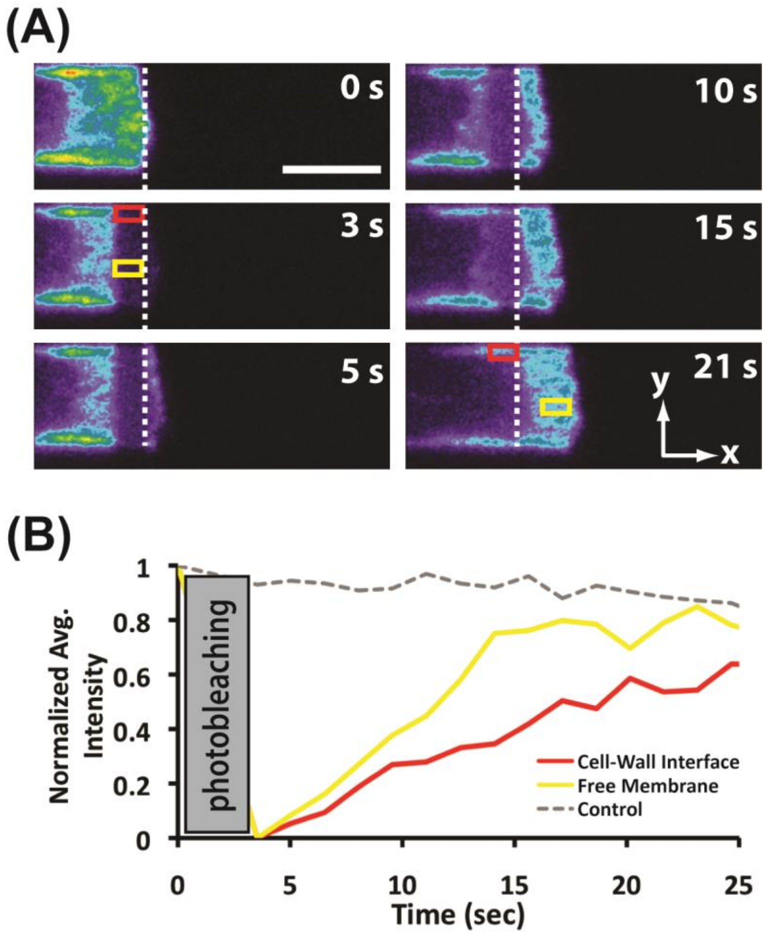

Supplementary Figure S5: Fluorescence recovery after photobleaching is faster at the free front membrane than at the cell-wall interface. In all images, cold colours indicate low fluorescence intensities, and hot colours denote high actin fluorescence intensities. Scale Bars = 5µm.

A. **FRAP experiment for a region including the free membrane at the front of a migrating cell expressing GFP-Actin** (same experiment as shown in **Fig 2A**). Photobleaching was carried out as shown on **Fig 2A**. In all panels, the white dashed line indicates the position of the free front membrane at time *t*=0s.
B. **Time course of fluorescence recovery after photobleaching.** Fluorescence recovery was monitored in two distinct regions: a photobleached region at the cell-wall interface (red line and red box in **A**) and a region following the free front membrane (yellow line and yellow box in **A**). Both regions were identical in size. The initial position for both regions is shown for *t*=3s in **A** and the final position is shown for *t*=21s in **A**. The region at the cell-wall interface reported on new polymerisation against the channel wall, while the region following the free front membrane reported on new polymerisation at the cell front. Fluorescence loss due to image acquisition was estimated in a ROI away from the bleach zone (grey line, “control”).

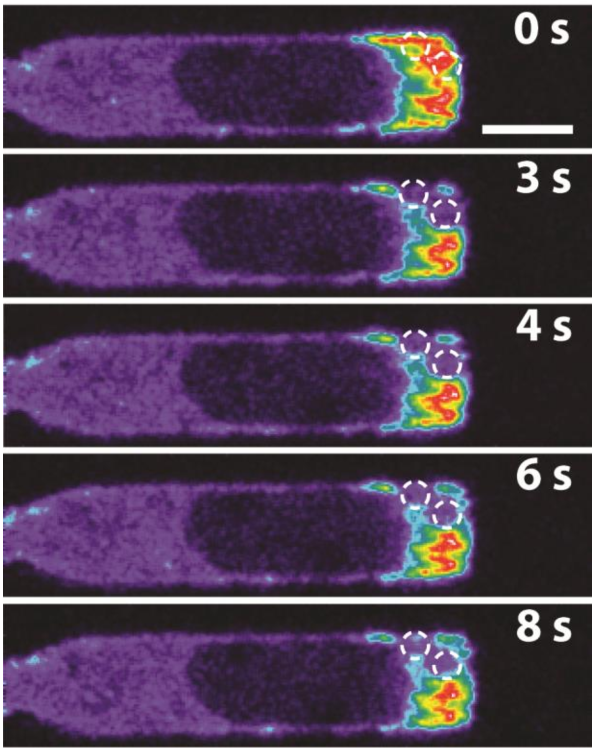

Supplementary Figure S6: Fluorescence recovery after photobleaching in two circular zones in the leading edge. One bleached region was located at the cell front and the other at the cell-wall interface. Following photobleaching, the bleached zones (dashed lines, *t*=0s) remained distinct and did not appear to fuse. This suggested that, despite the medial movement of the adherent F-actin network caused by new polymerisation against the cell-wall interface, the two networks retained their physical identity and did not exchange material over time. In all images, cold colours indicate low fluorescence intensities, and hot colours denote high actin fluorescence intensities. White indicates saturation in the chosen colour scale but does not reflect saturation during acquisition.

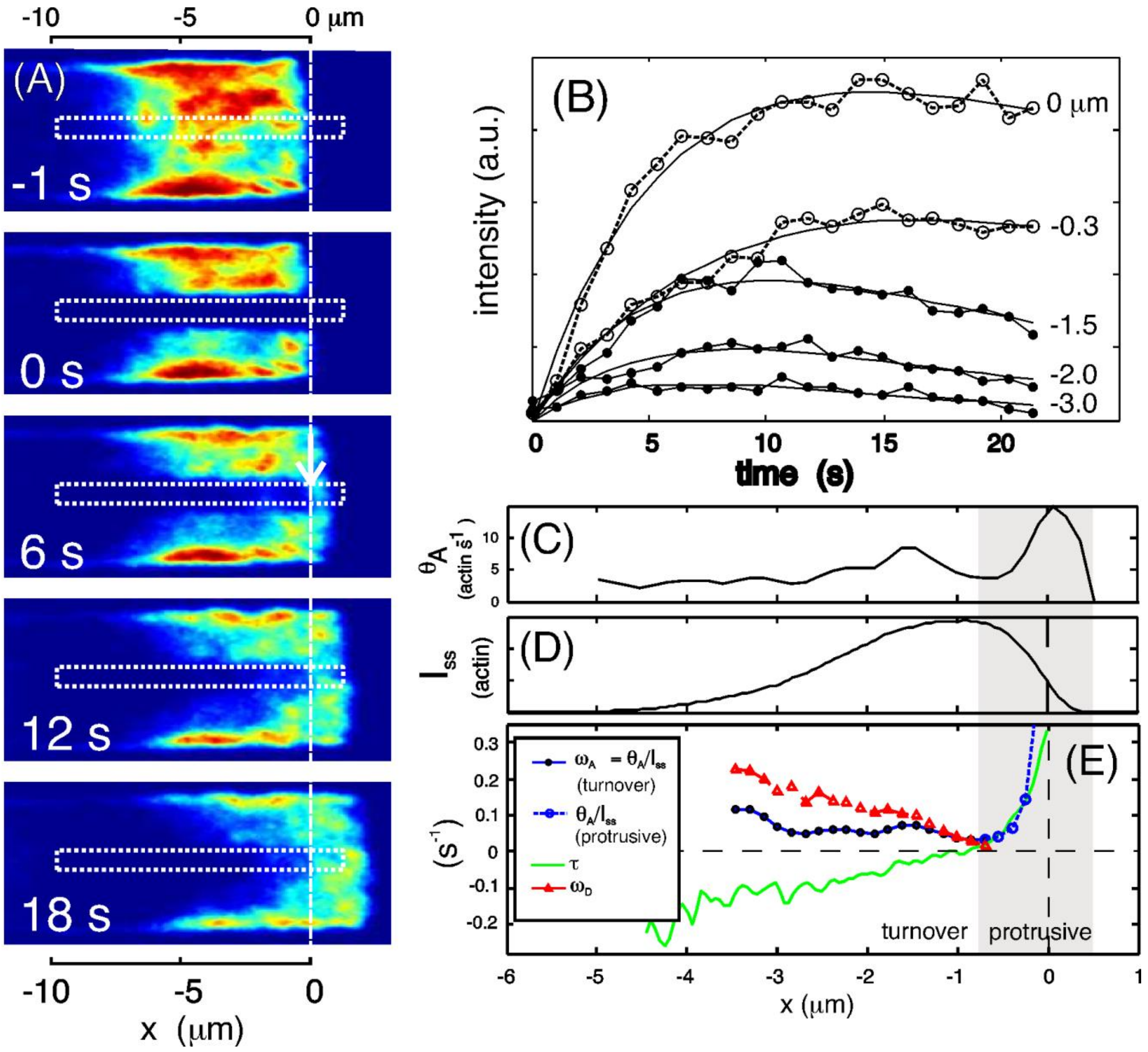

Supplementary Figure S7: Calculation of the assembly rate constant ω_A and the disassembly rate constant ω_D from FRAP experiments at the cell midline. In all images, cold colours indicate low fluorescence intensities, and hot colours denote high actin fluorescence intensities. In all panels, the position of the free membrane at the cell front at time *t*=0s was chosen as the origin along the *x*-axis.

A. **Time series of a FRAP experiment for a bleach region situated at the cell midline.** Photobleaching was performed in a 7μmx2μm rectangular region located at the cell midline spanning the whole leading edge length between times *t*=-2s and *t*=0s (red box, **Fig 3D**, **Supplementary Movie 5**). We monitored fluorescence recovery over time in an 8μmx1μm region (white box, **A**) narrower than the bleach region to minimise any potential contribution from F-actin network flows along the *y*-axis. This region was longer than the bleach region to allow calculation of fluorescence increase in regions of new protrusion (see arrow at *t*=6s). The position of the leading edge is indicated by the vertical dashed white line.
B. **Fluorescence increase as a function of time for different positions away from the free front membrane.** Change in fluorescence over time was monitored in contiguous 0.2μmx1μm regions within the white dotted box drawn in **A** choosing the position of the front membrane in the first frame after photobleaching as the reference along the *x*-axis (*t*=0s in **A**). All curves were qualitatively similar presenting first an increase in fluorescence intensity

followed by a plateau and then a slow decrease. Immediately behind the front membrane, fluorescence increase was due to new protrusion through insertion of G-actin monomers into existing filaments whose free barbed-ends abutted the front membrane as well as nucleation of new filaments (x=0µm and greyed area in **C-E**). For regions further away from the front membrane ($x \leq -0.5$µm), the initial increase was due to incorporation of fluorescent monomers into the bleached F-actin network. At longer time-scales, the decrease in intensity was likely due to a net decrease in the local F-actin density due to depolymerisation as the leading edge moved forward.

C. **Spatial profile of the rate of fluorescence increase along the cell midline.** Fitting of the experimental fluorescence increase curves shown in **B** with functions of the form $y(t)=a(1-e^{-bt})e^{-ct}$ (**B**, solid lines) allowed calculation of the spatial profile of the rate of fluorescence increase $\theta_A(x)$ (**Supplementary Methods**).

D. **Spatial profile of the steady state actin density along the cell midline.** The steady-state actin density along the midline $I_{SS}(x)$ was determined experimentally using images frames within the time-lapse sequence in which leading edge fluorescence had recovered entirely (**Supplementary Movie 5, Supplementary Methods**).

E. **Spatial profile of the assembly rate ω_A, disassembly rate ω_D, and net rate of change in F-actin density τ along the cell midline.** To compute apparent assembly rate constants, $\theta_A(x)$ was normalised to the steady-state F-actin density $I_{SS}(x)$ in each position x (blue curve). In regions of new protrusion (greyed region for 0µm$\geq x \geq$-0.5µm, dotted blue line and open symbols), $\theta_A(x)/I_{SS}(x)$ was due to nucleation of new filaments together with monomer incorporation at existing free barbed-ends abutting the front membrane. Elsewhere (for $x \leq$-0.5 µm, solid blue line and closed symbols), $\theta_A(x)/I_{SS}(x)$ reflected the apparent actin assembly rate constant ω_A for G-actin monomer incorporation into the free F-actin network through molecular mechanisms participating in turnover. As cells were at steady state and there was no retrograde flow, the spatial variation in F-actin intensity along the cell midline reflected spatial changes in the balance of F-actin assembly and disassembly and allowed computation of the net rate of change in F-actin density τ as $\tau(x) = -v[\partial I_{SS}(x)/\partial x]/I_{SS}(x)$ with v a constant reflecting the velocity of migration (green line). As the source term reflecting new protrusion was zero away from the membrane, the disassembly rate constant ω_D was then calculated as $\omega_D(x)= \omega_A(x) - \tau(x)$ (red line).

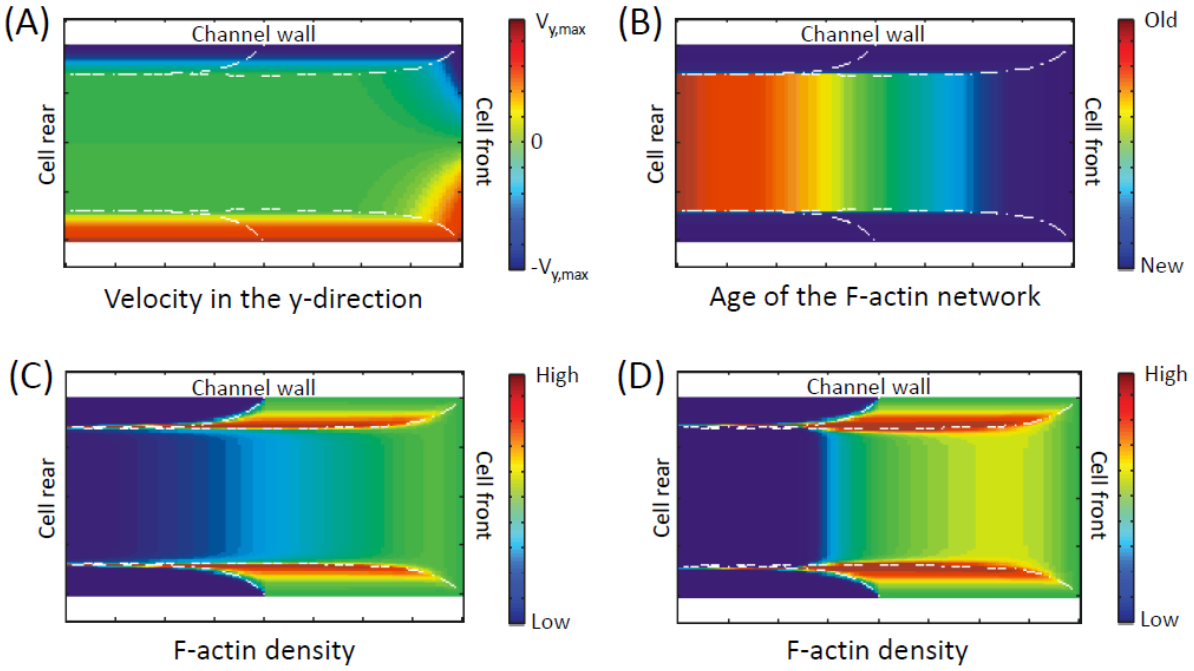

Supplementary Figure S8: Numerical simulation of F-actin density at the leading edge. Simulations were restricted to the leading edge only. New F-actin polymerisation took place at the cell front and the channel walls. The horizontal component of the F-actin network velocity (v_x) was taken constant throughout the leading edge in agreement with experimental data (**Fig 2C**). In all images, hot colours indicate high values and cold colours low values.

- **A.** Input velocity field in the vertical direction (v_y). The velocity field v_y was chosen based on the experimental measurements shown in **Figs 2-3** and **Supplementary Fig S3**.
- **B.** Age of the F-actin network throughout the leading edge based on flow velocities at boundaries and flow lines within the leading edge.
- **C.** F-actin density in the leading edge assuming that the depolymerisation rate ω_D did not depend on the age of the network (and hence the curvilinear distance *s* away from the cell membrane along flow lines).
- **D.** F-actin density in the leading edge assuming that the depolymerisation rate $\omega_D(s)$ did depend on the age of the network (and hence the curvilinear distance *s* away from the cell membrane along flow lines), in agreement with experimental data shown in **Fig 3G**.

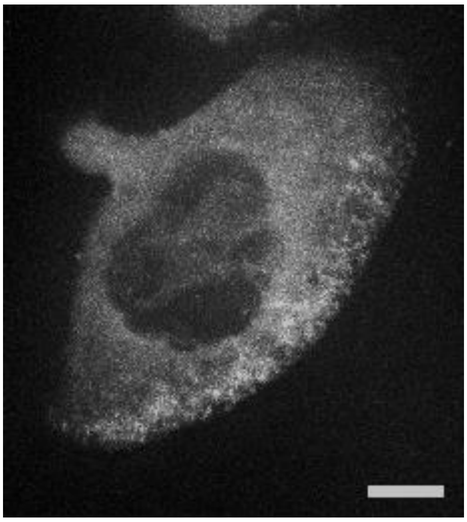

Supplementary Figure S9: The leading edge of neutrophils migrating on 2D substrates is enriched in the arp2/3 complex. The ARP3 subunit of the arp2/3 complex localised to the leading edge of a neutrophil migrating in 2-D. The direction of cell movement is towards the right. Scale bar = 5µm.

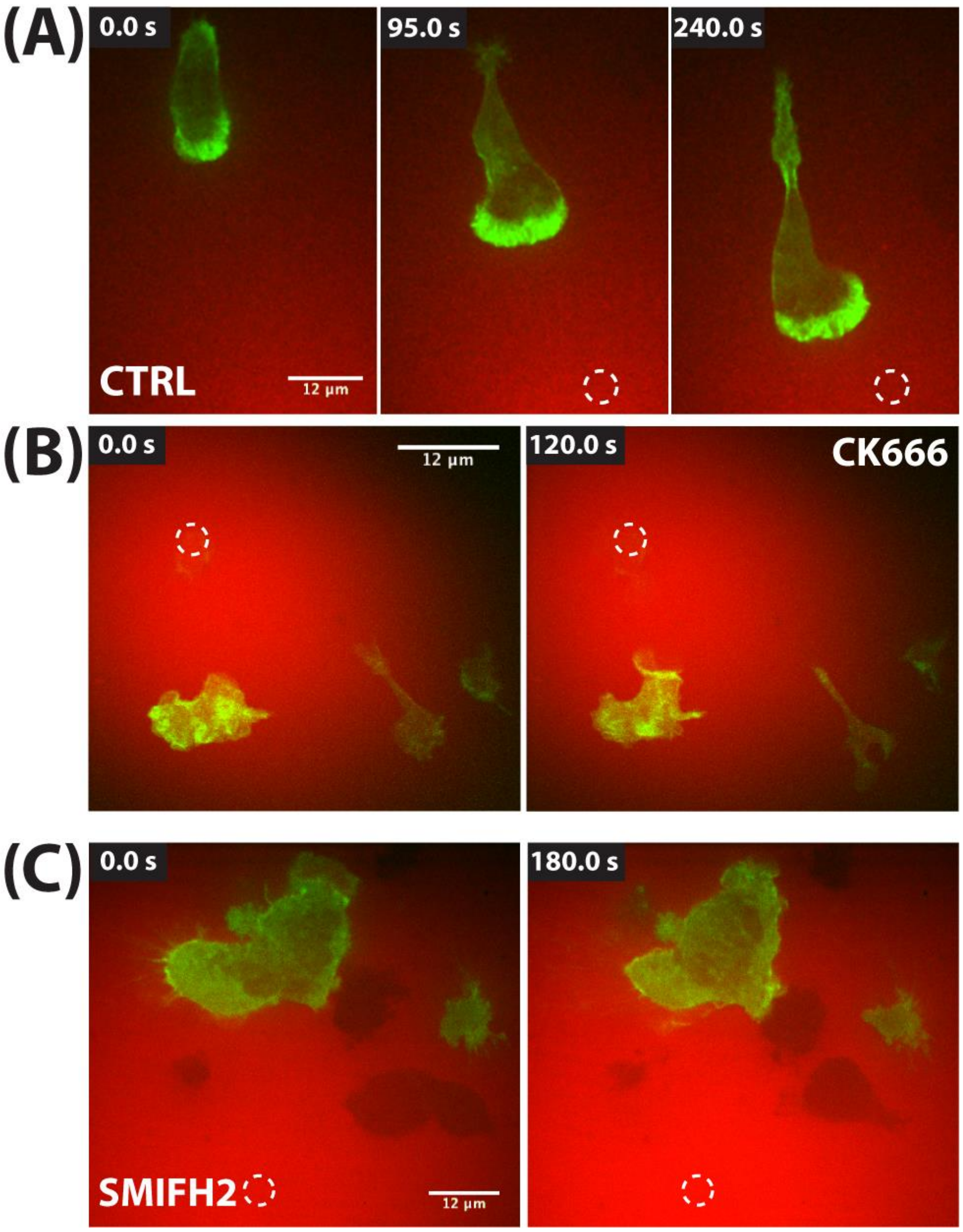

Supplementary Figure S10: Inhibition of the arp2/3 complex and formins blocks chemotaxis in 2-D.

In A, B, C, HL60 cells expressing GFP-Actin were exposed to a chemotactic gradient created by a micropipette slowly releasing 200µM fMLP. The micropipette tip is indicated by the dashed white circle and red fluorescence reflects the local chemoattractant concentration. Scale bars=12µm.

- **A.** Control cells moved rapidly towards the source of the chemoattractant gradient.
- **B.** Inclusion of CK666 in the imaging medium inhibited chemotaxis.
- **C.** Inclusion of SMIFH2 in the imaging medium inhibited chemotaxis.

Supplementary Methods

Microfluidic Device Fabrication

Masters for the microfluidic devices were fabricated using standard SU8 (MicroChem, Newton, MA, USA) photolithography. Three-inch single side polished silicon wafers were washed in isopropyl alcohol (IPA) and acetone, then baked on a hotplate at 150C for 5 minutes. A 5 µm layer of SU8-2005 was spun onto the wafer at 3000 RPM for 30 seconds. The wafer was baked at 65C for 1 min, then 95C for 3 min. The baked wafer was then placed in a Karl Suss MJB3 mask aligner and exposed for 2 seconds under a 4-inch by 4-inch chrome coated glass photomask (JD Phototools, Oldham, UK) printed with a negative image of the pattern for the migration channels. The wafer was then post-baked for 1 min at 65C, followed by 1 min at 95C. The first layer was then developed in EC solvent (Chestech, Rugby, UK) for one minute. After development, the wafer was washed with IPA and blown dry under a stream of nitrogen. A second layer of SU8-2025 was then spun at 2000 RPM to yield a ~40 µm thick layer. The wafer was baked at 65C for 3 minutes, followed by 7 minutes at 95C. The baked wafer was then exposed for 7 seconds under a 4-inch by 4-inch chrome-coated glass photomask printed with the pattern for the remainder of the microfluidic device. This was followed by development in EC solvent for 5 minutes or until all remaining uncrosslinked SU8 was removed. The wafer was then washed with IPA and dried under a stream of compressed ultrapure nitrogen.

Prior to casting of PDMS, the patterned wafers were immersed in a 1.0% solution of 1,1,2,2-perfluorooctyl trichlorosilane (13F, Fluorochem, Hadfield, UK) in toluene to prevent adhesion of the elastomer. A 10:1 mixture of Sylgard 184 prepolymer and initiator was thoroughly mixed and degassed prior to casting. The Sylgard/initiator mixture was carefully poured over the master and then baked at 75C for at least 4 hours. The cured PDMS was then carefully removed from the mould. Through-holes were punched for the inlets and outlet using a 1 mm biopsy punch (Kai medical, Honolulu, HI, USA). The PDMS devices were then treated for 30 seconds in oxygen plasma and bonded to 22 mm x 44 mm coverslips.

Chemical inhibitors

Cells were treated with cytochalasin D (500 nM to 1 µM, to cap actin filament barbed ends, Merck-Biosciences), CK666 (100 µM, to inhibit arp2/3 complex mediated F-actin nucleation[32], Merck-Biosciences), CK869 (100 µM, to inhibit arp2/3 complex mediated F-actin nucleation[32], Merck-Biosciences), and SMIFH2 (40 µM, to block formin mediated F-actin nucleation[35], Merk-Biosciences).

2D chemotaxis assays

For comparison with 3-D migration assays, some experiments were performed on Fibronectin (FN) coated glass coverslips. Round 25 mm glass coverslips were cleaned in oxygen plasma for 30 seconds immediately prior to use. Coverslips were coated with a PBS solution containing 50 µg/ml FN, and allowed to incubate at 37C for 1 hour. They were then washed 3 times with fresh PBS solution and covered with 1 ml of HBSS/HSA containing 10^5 cells/ml. The cells were allowed to attach for 1 hour at 37C prior to imaging. To assay chemokinetic migration, the chemoattractant f-MLP was added to the imaging medium to a final concentration of 100nM. To assay chemotactic migration (**Supplementary Fig S10**), cells were presented with a 200 µM solution of f-MLP leaking out of a glass micropipette. An Alexa-fluor647 labelled dextran was added to the pipette solution to allow visualization of the extent of the chemoattractant gradient.

Image analysis

Images were analyzed using Metamorph (Molecular Devices), ImageJ (http://rsbweb.nih.gov/ij/), iQ (Andor), and custom-written Matlab software.

Cell velocity and retrograde flow measurements were made using custom written programs using Matlab and the Matlab image analysis toolbox (Matlab, Natick, MA, USA).

Front to rear enrichment in cells expressing ARP3 and ARPC4 (**Fig 4E**) was computed by measuring the average fluorescence intensity in two zones of identical size: one at the front of the cell and the other at the rear of the cell. A ratio was then computed.

Actin fluorescence intensity enrichment in the adherent F-actin network (**Fig 5C**) was computed by measuring the fluorescence intensity profile across the cell width along a line ~2µm behind the free front membrane (**Fig 5B**). The adherent F-actin intensity was chosen to be the average value of the two peaks apparent at the cell-wall interface and this was normalised to the average fluorescence intensity in the centre of the profile where the free F-actin network was situated.

FRAP and iFRAP experiments

Manual analysis of fluorescence recovery and bleach zone aspect ratio

FRAP and iFRAP data were analysed using Metamorph software to output the fluorescence of chosen regions of interest (ROI). In experiments where the bleached zone moved within the framework of the laboratory, an ROI was drawn over the bleached zone in the first frame, its fluorescence intensity was measured, and it was manually moved to its position in subsequent frames. Following photobleaching, fluorescence in the ROI can recover either through diffusion of unbound fluorescent G-actin monomers (diffusive recovery) or through incorporation of fluorescent G-actin monomers into the F-actin network (reactive recovery). Recovery through diffusion is fast necessitating less than 250ms for regions with their smallest dimension equal to 1µm (see [53]). In our experiments, the first frame of recovery was collected 1s after completion of photobleaching therefore diffusive recovery was complete. Hence, by subtracting the average fluorescence intensity of the ROI in the first recovery frame from subsequent data points, we could monitor reactive recovery alone. In some measurements, fluorescence intensities were then normalised to the initial fluorescence intensity minus the cytoplasmic background (**Fig 2B,E**) and recovery curves were plotted using Microsoft Excel. To estimate fluorescence loss due to image acquisition, we measured the evolution of fluorescence intensity in control regions away from the ROI, subtracted background fluorescence intensity, and normalised their intensity to its initial value (**Fig 2B,E**).

Changes in aspect ratio of the bleached zone (**Fig 3A-C**) were assessed by manually drawing its shape in each frame of the movie to be analysed.

Image display

For better visualisation of fluorescence evolution at short times after photobleaching, time series are presented in pseudo-colour scales to enable regions of weak fluorescence to be clearly distinguished (**Fig 2, 3, Supplementary Fig S3-7**). In these images, pixels with intensities above the maximum in the chosen colour scale appear in white but this does not reflect saturation during acquisition of the fluorescence signal.

Generation of steady-state actin density maps and net change in actin density maps in the leading edge

In microchannels, cells migrated with a steady velocity and a highly constrained shape[13] allowing computation of the steady-state actin density distribution within the leading edge by time-averaging. Steady-state actin density maps $I_{SS}(x,y)$ were computed from time-lapse sequences of the actin distribution in the leading edge $i(x,y,t)$ such that $I_{SS}(x,y)=\langle i(x-vt,y,t)\rangle$ taking into account cell movement at a velocity v. In practice, we determined the position of the free membrane at the cell front at each time-point using intensity-based segmentation, reprojected the entire time-lapse sequence in the referential of the cell choosing the free membrane at the cell front as the origin along the x-axis, and computed the time-averaged actin fluorescence intensity at each (x,y) position within the leading edge (**Fig 1E**).

As cells moved forward in a steady-state during our experiments, the net instantaneous rate of change in fluorescence intensity at any given position within the leading edge was directly related to the spatial derivative of the actin distribution: $di(x,y,t)/dt = -v\, \partial i(x,y,t)/\partial x$ with v a constant equal to the velocity of migration. By averaging over all frames within the time-lapse, we can therefore define a time-averaged net instantaneous relative rate of change in fluorescence intensity as $\tau(x,y)=-v[\partial I_{SS}(x,y)/\partial x]/I_{SS}(x,y)$. Therefore, for each time-point and each position within the leading edge, we computed the steady-state actin distribution $I_{SS}(x,y)$, then $\partial_{SS}(x,y)/\partial x$ in the rest frame of a cell displaying steady-state motion, and normalised it to the steady-state actin fluorescence intensity at that position (**Fig 1F**).

Computation of polymerisation velocity at the cell boundaries in the leading edge

We calculated the x-component of the polymerisation velocity underlying protrusion at the leading edge by photobleaching a rectangular region covering the entire width of the cell front and then measuring the increase in width of the fluorescent actin band that formed just outside of the bleached region due to new protrusion created by actin polymerisation against the free membrane at the cell front (**Supplementary Fig S4A**). We identified the inner and outer boundaries of this band of newly polymerised actin using image segmentation by choosing a suitable threshold above the background intensity (see δx, **Supplementary Fig S4A**, t=10s). As shown in **Supplementary Fig S4B**, at each y-position the inner boundary of this band of fluorescence is essentially stationary, consistent with experiments showing lack of retrograde flow along the x-axis. In contrast, the outer edge (oriented in the direction of motion) advanced linearly over time reflecting new protrusion. We then calculated the velocity at each y-position across the channel as $v_x(y)= d(x_{outer}(y)- x_{inner}(y))/dt$ (**Supplementary Fig S4C**).

To compute the velocity profile $v_y(x)$ along the cell-wall interface, we measured the rate of medial movement of the adherent F-actin network in iFRAP experiments where the adherent network can easily be segmented from background using similar techniques to those described to measure v_x (**Supplementary Fig S3B**). In iFRAP experiments, the entire cell was photobleached except for a rectangular region comprising the adherent network (**Supplementary Fig S3B**). Thereafter, polymerization of non-fluorescent actin against the cell-wall interface displaced the fluorescent adherent F-actin network medially (**Supplementary Fig S3D**). Using segmentation based on fluorescence intensity, we detected the position of the external edge of the adherent F-actin network at all time points (**Supplementary Fig S4D**, white line). This allowed us to plot the position of the adherent F-actin network at regular x-intervals over time (**Supplementary Fig S4E**). At all x-positions, the y-position increased linearly over time (**Supplementary Fig S4E**) and we calculated the velocity profile at each x-position from the slope of each trajectory (**Supplementary Fig S4F**).

Computation of assembly and disassembly rate constants from FRAP experiments in the leading edge

For analysis of our experimental data, we formally defined the net rate of change in F-actin density τ as $\tau(x)= \omega_A(x)-\omega_D(x)+s\delta(x)$ with ω_A the F-actin assembly rate constant due to turnover processes, ω_D the F-actin disassembly rate constant, $s\delta(x)$ a source term with s its amplitude, and $\delta(x)$ the Dirac function. In our analysis, the rate constants ω_A and ω_D are only defined in regions where the initial fluorescence intensity prior to photobleaching is non zero. The last term reflects new protrusion generated by F-actin nucleation and elongation of existing filaments abutting the front membrane through G-actin incorporation at their barbed-ends. In the absence of density changes due to the convergence of actin network flow lines, ω_A and ω_D represent the local actin assembly and disassembly rate constants expressed in units of G-actin monomers incorporated per F-actin monomer in the network per second. Experimental measurements showed that there was no F-actin network flow relative to the substrate along the x-axis and, by symmetry arguments, there should be no flow convergence along the y-axis at the cell midline. Furthermore, to avoid any potential out of plane contributions to fluorescence recovery due to flows of F-actin network along the z-axis,

photobleaching experiments were effected in an *xy* plane midway through the height of the channel, where by symmetry arguments no flow convergence should take place.

To estimate $\tau(x)$, $\omega_A(x)$, and $\omega_D(x)$ from experiments, we first determined the local rate of fluorescence increase $\theta_A(x)$ from the initial slope of fluorescence recovery after photobleaching in a 7μmx2μm rectangle along the cell midline (**Supplementary Fig S7A** and red box**, Fig 3D**). $\theta_A(x)$ is expressed in units of fluorescence intensity per second reporting on the number of monomers of G-actin incorporated per second at each location *x*. In our analysis, the position of the free front membrane at *t*=0s, just after photobleaching, was chosen as the reference along the *x*-axis (vertical dashed white line, **Supplementary Fig S7A**). In zones of new protrusion (0μm>*x*>-0.5μm), θ_A reflected the rate at which G-actin monomers were inserted into existing filaments with their free barbed-ends abutting the front membrane and into newly nucleated filaments. In zones where an F-actin network was present prior to photobleaching (*x*<-0.5μm), during the first few seconds of recovery, fluorescence increase reflected only incorporation of new actin monomers into existing filaments in the region of interest because depolymerisation only removed bleached monomers. θ_A therefore reflected the rate at which G-actin monomers were inserted into existing filaments through the molecular processes underlying F-actin turnover.

In our experiments, we measured the rate of fluorescence increase θ_A in an 8μmx1μm region, narrower than the bleached region to minimise any potential contributions due to network flow but longer than the initial bleach region to allow determination of θ_A in areas of new protrusion (white box, **Supplementary Fig S7A**). The 8μmx1μm region was subdivided into contiguous rectangles of identical sizes centered at regular *x*-intervals and we measured actin fluorescence increase in each of these rectangles (a sample set of curves is shown in **Supplementary Fig S7B**). To exclude contributions due to diffusion of G-actin (see *manual analysis of fluorescence recovery*), we subtracted the average fluorescence intensity measured in each ROI in the first frame after photobleaching from subsequent data points. Each fluorescence increase curve was fitted to a function of the form $y(t)=a(1-e^{-bt})e^{-ct}$, where the slow decay term e^{-ct} accounts for loss of F-actin network density due to depolymerisation within the leading edge (solid lines, **Supplementary Fig S7B**). The rate of fluorescence increase $\theta_A(x)$ was taken as the absolute assembly rate *ab* derived from these fits at each position *x* (**Supplementary Fig S7C**). As expected, the rate of fluorescence increase θ_A was maximal at the free front membrane (**Supplementary Fig S7C**). Due to the limited spatial and temporal resolution of optical microscopy, the contribution of the source term $s\delta(x)$ to $\theta_A(x)$ in zones of new protrusion appeared as a bell-shaped curve spreading over ~1μm between *x*=[-0.5μm,0.5μm] and with a maximum value 3-4 fold larger than in neighbouring regions located for *x*<-0.5μm where turnover occurred (**Supplementary Fig S7C**). To compute apparent assembly rate constants, $\theta_A(x)$ was normalised to the steady-state F-actin density $I_{SS}(x)$ in each position *x* (**Supplementary Fig S7D**). In regions of new protrusion (greyed zone for *x*=[-0.5μm;0μm], dotted blue line and open symbols, **Supplementary Fig S7E**), $\theta_A(x)/I_{SS}(x)$ was due to nucleation of new filaments together with monomer incorporation at free barbed-ends abutting the front membrane. Elsewhere (for *x*<-0.5 μm, solid blue line and closed symbols, **Supplementary Fig S7E**), $\omega_A(x)=\theta_A(x)/I_{SS}(x)$ reflected the apparent actin assembly rate constant for G-actin monomer incorporation into the free F-actin network through molecular mechanisms participating in turnover. As in our formalism the rate constants ω_A and ω_D reflect assembly and disassembly due to turnover mechanisms, they are only defined in regions where the fluorescence intensity prior to photobleaching is non zero.

To minimise any potential effects due to reduction of the fluorescent G-actin monomer pool by photobleaching, we determined the steady state intensity profile along the midline $I_{SS}(x)$ using images frames within the time-lapse sequence in which the leading edge fluorescence had recovered entirely (**Supplementary Fig S7D**) using the same methods as described above (see *Generation of steady-state actin density maps*). The average steady-state actin density profile along

the x-axis enabled calculation of the net relative rate of change in actin density τ using the methods described above (**Supplementary Fig S7E**, green line) (see *Generation of steady-state actin density maps*). Finally, as the source term was zero away from the membrane, the spatial profile of the actin disassembly rate ω_D was calculated from the net rate of change in F-actin density τ and the assembly rate: $\omega_D = \omega_A - \tau$ (**Supplementary Fig S7E**, red line).

Numerical simulation of kinematic velocity maps

We performed a simple finite-element simulation to illustrate how a given polymerisation velocity field at the boundaries can reproduce the experimentally observed actin distributions. The actin network was modelled as a compressible fluid generated at the channel walls and at the front of the leading edge, while progressively disassembling throughout the leading edge with increasing distance away from the cell boundaries, in accordance with our experimental measurements (**Figs 2, 3**). This method of analysis allows exploration of a range of plausible velocity fields as well as potential kinetic models for the network decay of the steady-state density distribution. As we are searching for a steady state F-actin density distribution, we can assume that this distribution $I_{SS}(x,y)$ is stationary in the rest frame of the leading edge. We express the conservation of actin monomers as

$$\left(\frac{\partial I_{SS}}{\partial t}\right) = -\nabla.(I_{SS}v) - \omega_D I_{SS} = 0 \qquad \text{(Eq. S1)}$$

Where v is the local velocity and ω_D represents the rate of network decay (or disassembly) through depolymerization. The quantity $I_{SS}v$ represents the local flux of F-actin. The equation was solved by a standard finite-element matrix inversion in Matlab (Mathworks, Natick, MA, USA), assuming uniform distributions of F-actin nucleators along the cell-wall interface and at the free membrane at the front of the leading-edge, each constantly producing new F-actin. Consistent with experimental observations (**Fig 2C**), the velocity component v_x at the free membrane was set to a constant. We found empirically that a simple piecewise linear function for the velocity v_y in the orthogonal direction (i.e., towards the channel walls, see **Supplementary Fig S8A**) produced reasonable final results and were supported by our experimental observations (**Fig 2F, Supplementary Fig 3A**). Solving the equation in a Cartesian geometry (corresponding to an infinitely broad channel) or in a cylindrically symmetrical geometry yielded qualitatively very similar results, as the adherent F-actin network is very thin compared to the whole channel width. The rapid decay of v_y at the edges, which reflects medial growth of the adherent network leading to compression of the free F-actin network, produced the experimentally observed strong enhancement of F-actin density I at the interface between the two actin networks, and the more modest enhancement of the free F-actin network density in the bulk of the channel approximately 2µm behind the front membrane (compare **Supplementary Fig S8C,D** to **Fig 1E**).

Initially, we chose the rate of network disassembly ω_D as a constant, something equivalent to assuming that monomers embedded in the network dissociate and return to the G-actin pool with equal probability. This simple assumption was unable to produce the sharp front to rear gradient in actin density observed experimentally in the free actin network, with I_{SS} decaying too smoothly with distance from the front membrane along the central channel axis compared to experimental observations (compare **Supplementary Fig S8C** to **Fig 1E**). Experimental measurements of the spatial profile of the network disassembly rate ω_D showed that it increased with distance from the front membrane (**Fig 3G**). Therefore, we implemented a disassembly rate ω_D dependent on the time t elapsed since polymerization (i.e. the older the F-actin, the more likely its depolymerization). This was equivalent to choosing ω_D dependent on the curvilinear distance s away from the cell membrane along actin network flow lines. We calculated this “age function” at every point by integrating the velocity field (**Supplementary Fig S8B**). Choosing a rapidly increasing function for

$\omega_D(t)$ yielded results more qualitatively similar to the observations (Compare **Supplementary Fig S8D and Fig 1E**).

Whereas this analysis determined the actin density based on the compression of the actin network for an assumed velocity field, the true velocity field is likely to result from the balance of density-dependent compressive (and possibly shear) forces within the leading edge actin network. The local microscopic structure of the network may also play a significant role. A self-consistent solution of that problem is outside the scope of the present work. However, we believe our limited analysis reproduces the main qualitative features of the actin density distribution.
